# Distribution and Polymorphisms of Group I Introns in Mitochondrial Genes from *Cryptococcus neoformans* and *Cryptococcus gattii*

**DOI:** 10.3390/jof9060629

**Published:** 2023-05-30

**Authors:** Ronald Muryellison Oliveira da Silva Gomes, Kássia Jéssica Galdino da Silva, Leonardo Capistrano Ferreira, Thales Domingos Arantes, Raquel Cordeiro Theodoro

**Affiliations:** 1 Institute of Tropical Medicine, Universidade Federal do Rio Grande do Norte, Natal 59064-741, RN, Brazil; ronald.muryellison@gmail.com (R.M.O.d.S.G.); galdinokj@gmail.com (K.J.G.d.S.); ferreiralc@cb.ufrn.br (L.C.F.); 2 Department of Biochemistry, Center of Bioscience, Universidade Federal do Rio Grande do Norte, Natal 59064-741, RN, Brazil; 3 Institute of Tropical Pathology and Public Health, Universidade Federal de Goiás, Goiânia 74605-050, GO, Brazil; thales_domingos@ufg.br; 4 Department of Cell Biology and Genetics, Center of Bioscience, Universidade Federal do Rio Grande do Norte, Natal 59064-741, RN, Brazil

**Keywords:** cryptococcosis, autocatalytic introns, cryptic species, *cob*, *cox1*

## Abstract

The species complexes *Cryptococcus neoformans* and *Cryptococcus gattii* are the causative agents of cryptococcosis. Virulence and susceptibility to antifungals may vary within each species according to the fungal genotype. Therefore, specific and easily accessible molecular markers are required to distinguish cryptic species and/or genotypes. Group I introns are potential markers for this purpose because they are polymorphic concerning their presence and sequence. Therefore, in this study, we evaluated the presence of group I introns in the mitochondrial genes *cob* and *cox1* in different *Cryptococcus* isolates. Additionally, the origin, distribution, and evolution of these introns were investigated by phylogenetic analyses, including previously sequenced introns for the *mtLSU* gene. Approximately 80.5% of the 36 sequenced introns presented homing endonucleases, and phylogenetic analyses revealed that introns occupying the same insertion site form monophyletic clades. This suggests that they likely share a common ancestor that invaded the site prior to species divergence. There was only one case of heterologous invasion, probably through horizontal transfer to *C. decagattii* (VGIV genotype) from another fungal species. Our results showed that the *C. neoformans* complex has fewer introns compared to *C. gattii.* Additionally, there is significant polymorphism in the presence and size of these elements, both among and within genotypes. As a result, it is impossible to differentiate the cryptic species using a single intron. However, it was possible to differentiate among genotypes within each species complex, by combining PCRs of *mtLSU* and *cox1* introns, for *C. neoformans* species, and *mtLSU* and *cob* introns for *C. gattii* species.

## 1. Introduction

Cryptococcosis is a systemic/opportunistic mycosis caused by pathogenic basidiomycete yeasts from the species complexes *Cryptococcus neoformans* and *Cryptococcus gattii*, which in nature can be found in different environments, such as bird excrement (especially from pigeons), soil, rivers, decomposing vegetation, and tree hollows [1,2]. The most common forms of cryptococcosis are pulmonary and central nervous system (CNS) infections, with meningitis as the most frequent and severe manifestation. In 2020, approximately 152,000 new cases of cryptococcosis associated with AIDS were reported worldwide, resulting in an estimated 112,000 deaths [3].

Regarding *Cryptococcus* species, the necessity of more precise techniques for the diagnosis has led to the development of some molecular approaches, such as AFLP and PCR-RFLP of the *URA5* gene, to encompass all genetic variability of clinical and environmental isolates [4,5]. The genotypes proposed by these techniques were organized in at least seven cryptic species by a multi-locus sequencing typing study using eleven genetic loci [6]: *C. neoformans* (genotypes VNI, VNII, VNB), *C. deneoformans* (VNIV), *C. gattii* (VGI), *C. deuterogattii* (VGII), *C. bacillosporus* (VGIII), *C. tetragattii* (VGIV), and *C. decagattii* (VGVI). The VNIII genotype is believed to be a hybrid between *C. neoformans* and *C. deneoformans*. Recently, Farrer et al. [7] reported a genetically distinct lineage of *C. gattii* in forests of Zambia, in association with nitrogen-rich excreta from a small mammal, known as Hyrax (*Dendrohyrax arboreus*). This new genotype was named VGV.

From a clinical perspective, accurately identifying the species or genotype of *C. neoformans* and *C. gattii* isolates is of utmost importance, as there are variations in the susceptibility to antifungals among different genotypes. Clinical data suggest that the response to antifungal therapy is less responsive in *C. gattii* infections, requiring prolonged treatment, especially because they are associated with cryptococcomas formation [8,9,10]. Additionally, it has been found that the VGII molecular type (*C. deuterogattii*) is the least susceptible to antifungals (especially azoles), followed by VGI, VNI, and VNIV isolates [11,12,13,14].

Moreover, considering the cryptic speciation in the *C. neoformans* and *C. gattii* complexes, the quest for novel and readily accessible molecular markers for genotyping becomes highly essential, and group I introns appear to be well-suited for such investigations, in view of the fact that they are polymorphic in their presence and sequence [15]. Those elements are self-splicing RNAs that range in size from 250 to 500 nucleotides [16], but some of them can be larger than 3000 nucleotides, as they can present homing endonucleases genes (HEG) [17]. These HEGs are responsible for the mobility of the introns as they recognize and cleave the cognate allele (which lacks the intron), and through homologous recombination, the intron is copied to this new site. It is worth noting that, in some cases, the HEGs may exhibit “promiscuity” and recognize heterologous sites, thereby facilitating the spread of the intron to other locations [18]. Besides, their self-splicing, which is essential for fungi’s survival since it occurs in housekeeping genes, is known to be inhibited by some drugs [19,20,21,22,23]. This characteristic becomes increasingly relevant in light of the rising number of cases of opportunistic fungal diseases, which are clinically exacerbated due to the emerging resistance to antifungals [24].

In a recent study, our research team examined the presence of group I introns in the *mtLSU* gene, which encodes the large-subunit rRNA of the mitochondrial ribosome [25]. We found that genotypes without introns were those already known in the literature for being more virulent. To ascertain whether this pattern applies to other group I introns in the mitogenome of *C. neoformans* and *C. gattii,* and to assess whether these introns possess informative polymorphism for distinguishing among genotypes or species complexes, we investigated the presence, polymorphism, and phylogenetic relationships of group I introns in the mitochondrial genes *cox1* (cytochrome c oxidase) and *cob* (cytochrome b). These genes were selected because the current literature has demonstrated them to be the most frequently invaded by introns in fungal mitogenomes [26].

## 2. Materials and Methods

### 2.1. Fungal Isolates and Genotyping

Seventy-four of the seventy-six *Cryptococcus* isolates used in this study had already been genotyped by Gomes et al. [25] by PCR-RFLP of the *URA5* gene, as described by Trilles et al. [27]. Among these, 49 isolates are *Cryptococcus neoformans* (38 VNI, 5 VNIV, 3 VNII, 3 VNIII) and 25 are *Cryptococcus gattii* (19 VGII, 2 VGI, 2 VGIII, 2 VGIV). Two recent strains (HGT17 and HGT18) were isolated from patients hospitalized at Hospital Giselda Trigueiro in Natal, under the approval of the Research Ethics Committee of the Federal University of Rio Grande do Norte: CAAE 39640614.8.0000.5537, and they were genotyped in this study as mentioned above. The list of all isolates, their genotypes, and sources is available in Supplementary File S1.

### 2.2. PCR and Sequencing of the Group I Introns Present in the cob and cox1 Genes of Cryptococcus

**Figure 1:**
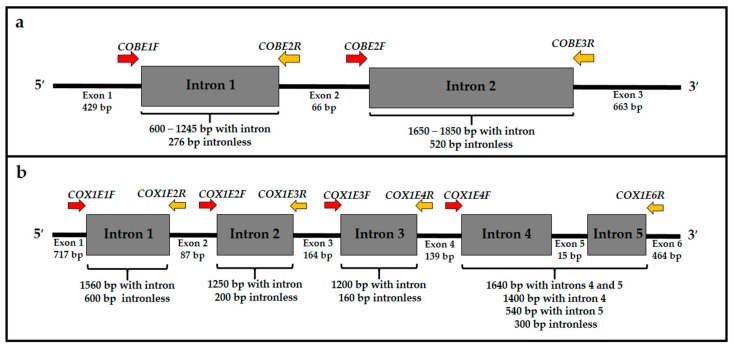
Scheme of the annealing regions of the primers designed for the amplification of group I introns from the *cob* (**a**) and *cox1* (**b**) genes. Red arrows indicate forward primers, while yellow arrows indicate reverse primers. The primers were designed based on the sequences described by Litter et al. [28].

PCRs of the group I introns from *cob* and *cox1* genes were performed using manually designed primers (Table 1) which align with the adjacent exons at a distance of 100 to 300 bp, on average, from the 5′ and 3′ ends of the intron (Figure 1). To design the primers, the reference sequences of *cox1* and *cob* genes from *Cryptococcus* [28], containing group I introns, were aligned using the MEGA X software (v.10.2) [29].

PCR reactions were performed with GoTaq Polymerase (Promega, Madison, WI, USA), according to the manufacturer’s instructions. The thermocycling consisted of an initial cycle of 30 s at 98 °C for initial denaturation, followed by 35 cycles of 10 s of denaturation at 98 °C, 30 s of annealing at 53 °C, 1 min of extension at 72 °C, and a final extension cycle of 10 min at 72 °C. Electrophoresis was performed in 1% agarose gel (GE Healthcare, Chicago, IL, USA) with ethidium bromide and visualized under a UV transilluminator (Loccus, Cotia, SP, Brazil). To confirm the presence of introns and endonuclease homing genes, some PCR products of different sizes, in the different genotypes, were selected and purified using the IlustraTM GFXTM PCR DNA and Gel Band Purification kit (GE Healthcare, Chicago, IL, USA), according to the manufacturer’s instructions, and subjected to capillary electrophoresis sequencing at MACROGEN/South Korea, in order to confirm the presence or absence of the intron.

### 2.3. Characterization of the Amplified Group I Introns and Search for Endonuclease Genes

As group I introns are divided into five classes, based on structural characteristics (IA-IE), the online tool RNAweasel [17] (https://megasun.bch.umontreal.ca/RNAweasel/), accessed on 20 January 2023, was used to classify the sequenced introns, according to their RNA secondary structure. The algorithm ERPIN [30] was used for the same purpose. The presence of endonuclease genes in these sequences was also mapped using the online tool Conserved Domain Database (https://www.ncbi.nlm.nih.gov/Structure/cdd/wrpsb.cgi), accessed on 25 January 2023.

### 2.4. Phylogenetic Analyses

Phylogenetic analyses were conducted on the *cox1* and *cob* introns, which were sequenced in this study, in addition to the *mtLSU* introns already described [25]. Three phylogenetic analyses were carried out: one just with *cob* introns, one just with *cox1* introns, and another one with introns from *mtLSU*, *cob*, and *cox1* genes. The aim was to investigate the possibility of the same intron having invaded different genes or different sites within the same gene, since heterologous invasions may occur under certain circumstances that decrease the homing endonuclease specificity, such as HEG mutations and oxidative stresses [18,31]. For this, all introns had the homing endonuclease domain removed from their sequences before they were submitted to alignment in the MEGA X program (v.10.2) by the clustalW method. The analysis was conducted by the Maximum Likelihood inference method, following the Kimura 2-parameter model [32]. For statistical confidence of the branches, a bootstrap with 1,000 replicates was used, with the percentages shown next to the branches. The tree was visualized in FigTree (v.1.4.2), and Inkscape (v.0.91) was used for final editing, preserving the scale.

### 2.5. Statistical Analyses

In order to test for the association between group I introns and species complexes, we used Fisher’s exact test (intron treated as categorical data: present or absent) or the Mann–Whitney test (intron treated as quantitative data: number of introns). The statistical analyses were performed using Stata software (v. 11.1) and the significance level was established at *p* < 0.05.

## 3. Results

### 3.1. Size and Sequence Polymorphism of the Group I Introns from Mitochondrial Genes cob and cox1 among Cryptococcus neoformans and Cryptococcus gattii Genotypes

**Figure 2:**
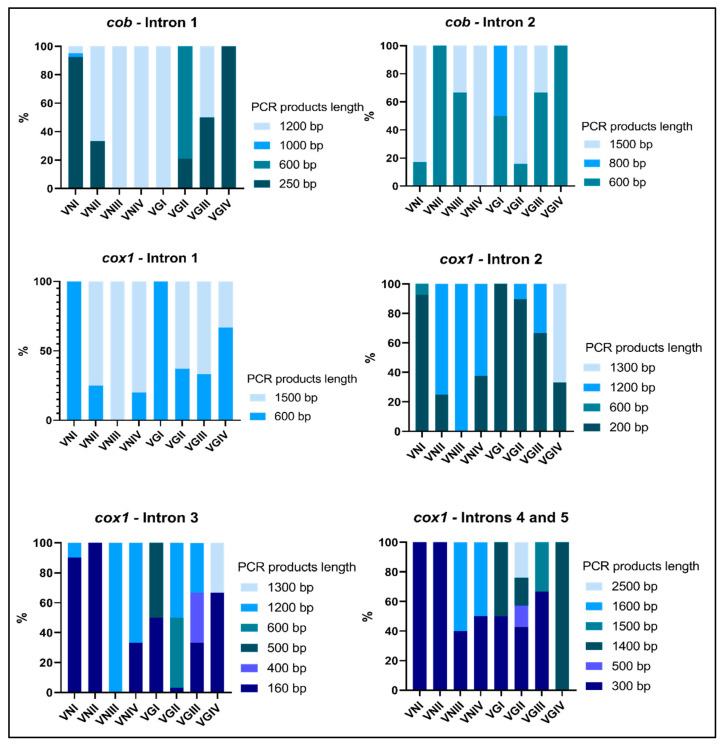
Frequency of different PCR sizes for the introns amplified in *cob* and *cox1* genes in different genotypes of *C. neoformans* and *C. gattii*.

According to PCR-RFLPs of the URA5 gene, the two new isolates from clinical samples (HGT 17 and HGT18) belong to the VNI genotype. The size of PCR products for group I introns from the *cob* gene are listed at the Supplementary File S2 (Table S1) and the variation on their size polymorphism is illustrated in Figure 2. Most of the VNI isolates and the two VGIV isolates evaluated do not present any intron in the first insertion site, showing a PCR product of 250 bp, which refers to the flanking exonic region, different from what was observed for the other genotypes of the *C. neoformans* and *C. gattii* complexes, whose PCR products ranged from 600 to 1200 bp. Regarding the intron in the second insertion site of the *cob* gene, its presence was confirmed by the amplification of a 1500 bp band for majority of the isolates from the different genotypes, with the exception of the VNII and VGIV isolates studied here, which presented a 500 bp band, which refers to the flanking exons. The two bands, 500 and 1500 bp, for the absence and presence, respectively, of the second intron from the *cob* gene, were amplified for one VGIII and one VNI isolate, indicating a possible heteroplasmy, i.e., the presence of different mitochondria in the same cell.

The size of PCR products for group I introns from the *cox1* gene for each fungal isolate are listed at the Supplementary File S2 (Table S2). Their size polymorphisms are illustrated in Figure 2. Concerning the first intron in the *cox1* gene, the 600 bp product (indicating its absence) was obtained for all VNI isolates. For the other genotypes, most of the isolates presented a 1500 bp band, indicating the presence of the intron. Some isolates (1 VNII, 8 VGII, 1 VGIII, and 1 VGIV) presented both bands, 600 bp and 1500 bp. The second intron was absent in almost all VNI and VGII isolates (200 bp band) and present in most of the isolates from the other genotypes (1200 bp band). Some isolates (1 VNII, 3 VNIV, 1 VGIII, and 1 VGIV) presented both bands: 200 bp and 1200 bp. The third intron was absent in almost all VNI isolates and in all VNII and VGIV isolates (160 bp band). PCR products indicating the presence of the third intron presented 1200 bp for some VNI, VNIII, VNIV, and VGII isolates, 400 bp for a VGIII isolate, and 500 bp for a VGI isolate. Some isolates (1 VNIV and 15 VGII) also presented two bands (160 and 1200 bp). Regarding introns 4 and 5, they were amplified together, in one single PCR reaction. For this PCR, the band of 300 bp, which refers to the flanking exons only, was observed for all VNI and VNII isolates and for a few of the isolates belonging to the other genotypes. Bands indicating intron presence had different sizes: >1600 bp for the presence of both introns 4 and 5, 1400–1500 bp for the presence of intron 4 only, and 500 bp indicating the presence of intron 5 only. Signs of heteroplasmy were also detected here, including isolates with the three possible bands. The presence of more than one band was observed for 1 VNIII, all VNIV, for 17 of the 19 VGII, and for 1 VGIII (Table S2 from Supplementary File S2).

The sequencing of some of the PCR products was conducted to compare their sequences to those used as references for primer design. The GenBank accession numbers for all the 36 sequenced introns are OQ946649 to OQ946684. One sequenced intron, from the FC9 isolate (VGIV), amplified with the primers flanking the second intron from *cox1*, was actually localized in a new insertion site, between the second and the third intron. A sequence similarity search was conducted using the online platform BLAST (Basic Local Alignment Search Tool) (https://blast.ncbi.nlm.nih.gov/Blast.cgi), accessed on 10 March 2023, which revealed that this intron shares significant similarities with introns found in other fungi. For example, it exhibited 78% identity, with a coverage of 20% and an e-value of 2 × 10^−16^, when compared to the fourth intron in the *cox1* gene from *Arthonia susa*, a lichenized ascomycete. Furthermore, it demonstrated 74.73% similarity, with an 18% coverage and an e-value of 4 × 10^−19^, when compared to the fourth *cox1* intron from *Phellinotus piptadeniae*, a wood-decay basidiomycete. Additionally, it showed 74.48% similarity, with a coverage of 31% and an e-value of 5 × 10^−18^, when compared to a nuclear sequence on chromosome 7 from *Apiotrichum mycotoxinovorans* (*Trichosporon mycotoxinovorans*), an opportunistic basidiomycete pathogen.

For sequences containing introns, we observed size and sequence polymorphisms that were associated with the presence of open reading frames (ORFs) containing homing endonuclease genes (HEGs), all from the LAGLIDADG family. These ORFs were detected in 29 of the 36 sequenced introns.

The primary sequence of each sequenced intron (available in Supplementary File S4) was used to classify them using the online tool RNAweasel [17]. Table 2 summarizes the classification of group I introns and indicates which ones have HEGs. The analysis showed that the *cox1* gene is only invaded by introns from the families IA and IB, while the *cob* gene only has introns from the families IA and ID.

### 3.2. Distribution of Group I Introns in the Mitogenome of Cryptococcus neoformans and Cryptococcus gattii

**Figure 3:**
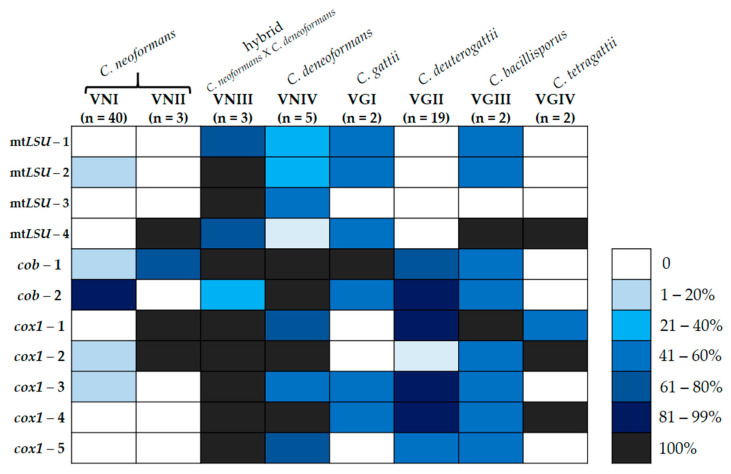
Distribution of group I introns in the *mtLSU*, *cox1,* and *cob* genes in *C. neoformans* and *C. gattii* species complexes. Frequency of isolates with group I introns in the different genotypes/species of each species complex.

**Figure 4:**
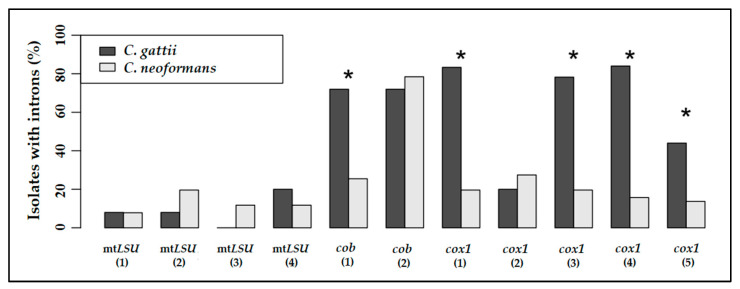
Frequency of isolates with group I introns at each insertion site, in both species complexes. * Bonferroni-corrected *p*-value < 0.05 (Fisher’s exact test).

The search for group I introns in the *cob* and *cox1* genes, conducted in this study as well as in *mtLSU* [25] on the same group of fungal isolates, allowed us to gain an overview of the distribution of those introns within the three mitochondrial genes for both species complexes, *C. neoformans* and *C. gattii*, as well as their genotypes (or cryptic species), as shown in Figure 3 and Figure 4.

Figure 3 shows that the genotype with the highest frequency of group I introns in the eleven sites analyzed here (four in *mtLSU*, two in *cob*, and five in *cox1*) was VNIII, a hybrid between the species *C. neoformans* and *C. deneofomans* (VNIV) [6], while the genotype with the lowest frequency of introns was VNI, considered the most virulent *C. neoformans* and the most commonly isolated genotype, from clinical specimens, in the world [33].

Regarding the distribution of group I introns between the *C. neoformans* and *C. gattii* complexes, there was no statistical significance between the amount of introns in the *mtLSU* gene, as previously mentioned [25]. However, our data showed an opposite situation for the introns in the *cob* and *cox1* genes, mainly for the intron 1 of *cob*, and the introns 1, 3, 4, and 5 of *cox1* (Figure 4).

**Figure 5:**
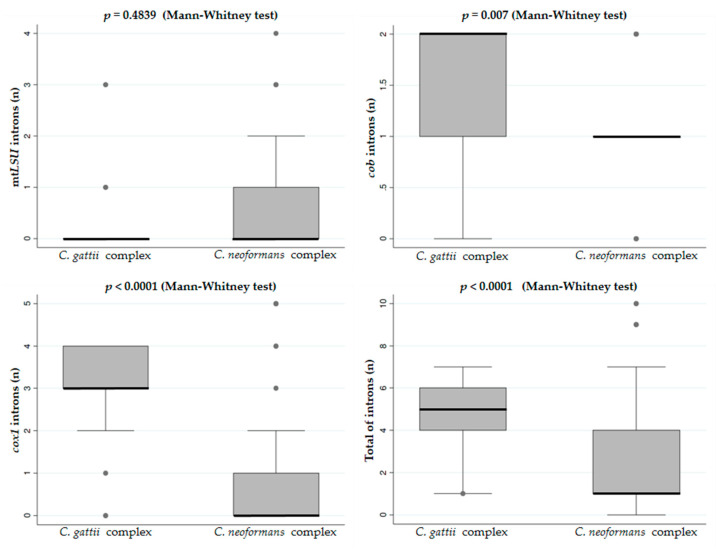
Number of group I introns in the *mtLSU*, *cob,* and *cox1* mitochondrial genes in *C. neoformans* and *C. gattii* species complexes. *C. gattii* has more introns in the *cob* and *cox1* genes. Statistical analyses were performed using Stata v.11.1.

For both *cob* and *cox1* genes, the *C. gattii* complex showed, on average, more introns than *C. neoformans*, and this pattern was maintained when all the introns from the three genes were analyzed together (Figure 5).

### 3.3. Phylogenetic Analyses

**Figure 6:**
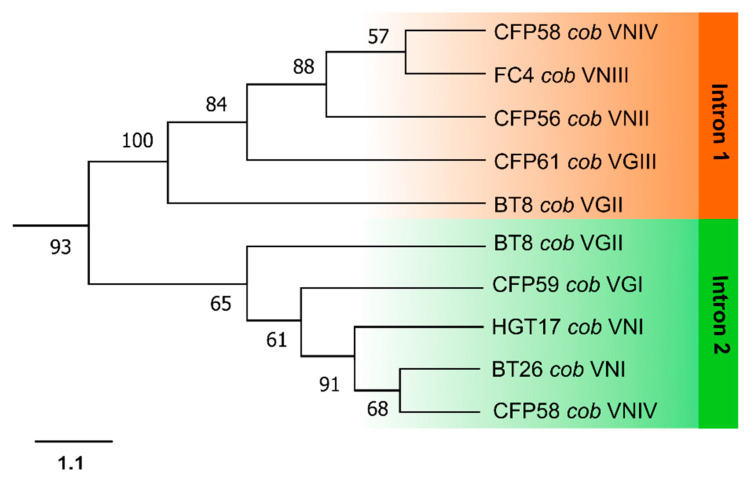
Phylogeny of *cob* introns from different isolates belonging to *C. neoformans* and *C. gattii species* complexes. Phylogenetic relationships were inferred by the Maximum Likelihood method in the Mega X program. The bootstrap values (%) are shown next to the branches.

**Figure 7:**
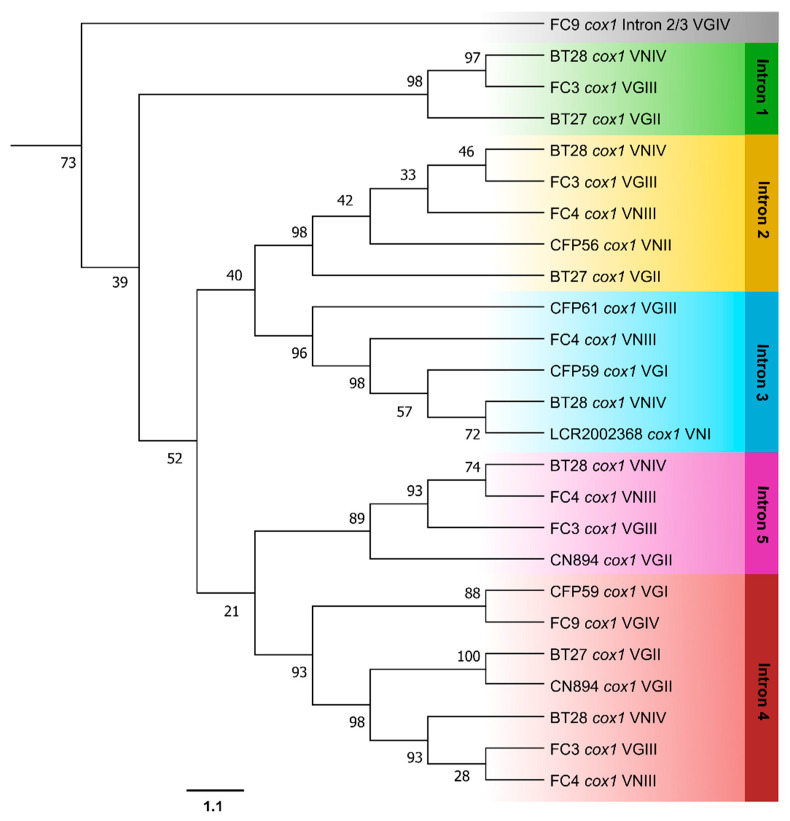
Phylogeny of *cox1* introns from different isolates belonging to *C. neoformans* and *C. gattii* species complexes. Phylogenetic relationships were inferred by the Maximum Likelihood method in the Mega X program. The bootstrap values (%) are shown next to the branches.

In both *cob* (Figure 6) and *cox1* (Figure 7) intron phylogenies, it was observed that introns occupying the same insertion site formed well-defined monophyletic clades. For example, in the phylogenetic analysis of *cob* introns, introns at the first insertion site showed closer relatedness to each other, while introns at the second insertion site displayed a similar pattern (Figure 6).

In the *cox1* gene, the introns at positions 2 and 3 exhibited a closer relationship to each other, as did the introns at positions 4 and 5. Interestingly, the intron found in a new position in the FC9 isolate (in *cox1*, between insertion sites 2 and 3) has been proven to be an outlier, considering its lower similarity to the other introns (Figure 7).

In both cladograms, it is evident that the *cob* and *cox1* intron sequences from various isolates of the *C. neoformans* and *C. gattii* complexes lack informative sites for distinguishing genotypes or cryptic species within those complexes. Even the complexes themselves cannot be separated, as some introns from *C. neoformans* are closer to introns from *C. gattii* than to others from *C. neoformans*, for example.

**Figure 8:**
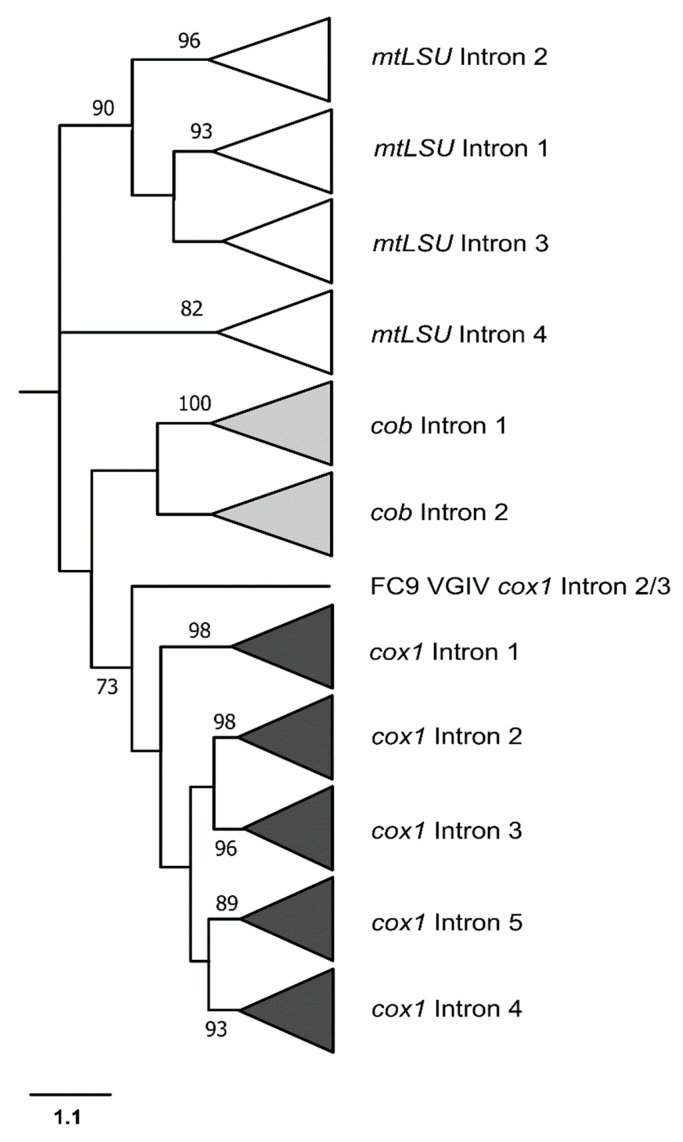
Phylogeny with all group I introns sequenced from *mtLSU*, *cob* and *cox1* genes, from different isolates belonging to *C. neoformans* and *C. gattii* species complexes. Introns occupying the same position form a monophyletic group, and therefore their branches were collapsed to represent the phylogenetic relationship among intronic sequences. Sequences of group I introns from the *mtLSU* gene were obtained from Gomes et al. [25]. The bootstrap values (%) are shown next to the branches.

With the addition of the *mtLSU* intronic sequences, it was possible to build a cladogram with the phylogenetic relationships of the introns from the three genes (Figure 8).

### 3.4. Proposal of PCR Reactions for Differentiation of Cryptococcus Species Based on Mitochondrial Group I Introns

**Figure 9:**
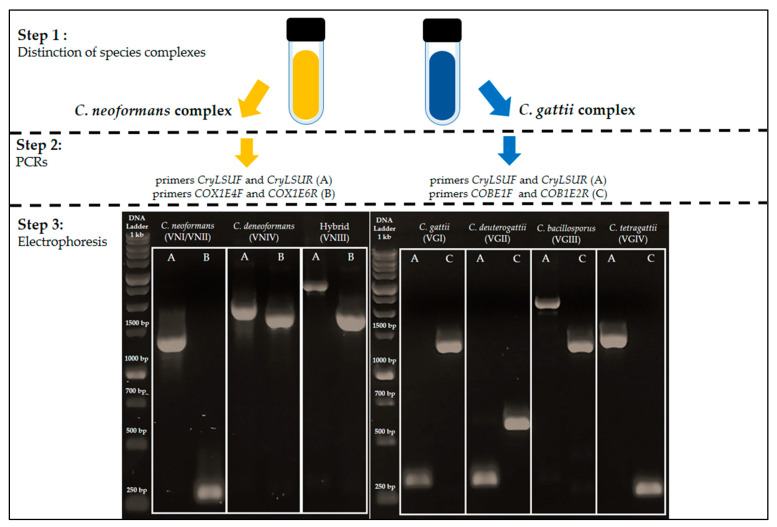
Gel electrophoresis showing the different genotypes/species of *C. neoformans* (left) and *C. gattii* (right). For *C. neoformans*, the bands on the left, within each rectangle, were obtained through PCR using manually designed primers, specifically *CryLSUF* (5′ GATTTGACTATTCTTATGTGC 3′) and *CryLSUR* (5′ GGTATATGCATGCTTGACTGC 3′), from Gomes et al. [25], for group I introns from the *mtLSU* gene (A), while the bands on the right, within each rectangle, were obtained with the cox4/5 primers (B) (Table 1 and Table 3). For *C. gattii*, the bands on the left, within each rectangle, were also generated with the *mtLSU* primers (A), and those on the right with *cob* primers (C) (Table 1 and Table 3). The DNA used was obtained from isolates BT12 (VNI), CN117 (VNIII), FC2 (VNIV), BT14 (VGI), BT17 (VGII), CFP61 (VGIII), and FC9 (VGIV).

The results obtained by PCR to determine the presence or absence of group I introns suggest that the occurrence of these elements in *Cryptococcus mitochondrial* genes may vary within each genotype. This indicates that there are not enough polymorphisms within different genotypes in order to use these introns as molecular markers in a differentiation scheme among genotypes through a single PCR reaction. However, it was possible to distinguish among species using two pairs of primers after determining the species complex to which a given isolate belongs, whether it is *C. neoformans* or *C. gattii*. Therefore, based on the distribution and size of group I introns in the mitochondrial genes *mtLSU*, *cox1*, and *cob*, we proposed a primer scheme that differentiates between the species described by Hagen et al. [6], following a previous differentiation between the complexes in culture medium (CGB agar) [34], or by singleplex PCR [35] or biochemical tests [36]. Table 3 below summarizes which primers should be used in this scheme and Figure 9 shows their amplification band patterns.

The similarity between band patterns of different species complexes can be observed in the example shown in Figure 9. The band patterns observed for *C. neoformans* were very similar to those observed for *C. tetragattii*, reaffirming the need to differentiate between the complexes prior to PCR reactions.

## 4. Discussion

Since the discovery that cryptococcosis can be caused by various species from the *C. neoformans* and *C. gattii* complexes, species-specific identification has become crucial. Genetic differences between these species are likely to affect aspects of diagnosis, pathogenicity, and treatment. Therefore, this study aimed to investigate the distribution of mitochondrial group I introns in the genes *mtLSU*, *cox1*, and *cob* in pathogenic species of *Cryptococcus* and to determine whether these genetic elements can serve as markers for species or genotype differentiation.

The PCR results confirmed that the presence or absence of self-splicing introns in the mitochondrial genes of *Cryptococcus* varies within each complex and genotype, likely due to recurrent losses and gains of introns as a result of the homing process. A recent study analyzed 184 mitogenomes from different genotypes of *C. neoformans* (VNI, VNII, and VNB) and detected variations in size ranging from 24,740 to 31,327 bp, primarily due to differences in the number and size of mitochondrial introns, as well as the presence of HEGs [37].

Another noteworthy finding is that, on average, the *C. neoformans* complex had fewer group I introns than *C. gattii* (*p* < 0.001) in the *cox1* and *cob* genes. This represents a distinct pattern from what has been previously observed for the introns in the *mtLSU* gene [25]. For instance, the VGII genotype, considered the most virulent, did not have any introns in the *mtLSU* gene, although it had a high frequency of introns in the *cox1* and *cob* genes. The association between intron presence and virulence requires further investigation for introns in different mitochondrial genes through an experimental approach, using animal models.

One possible explanation for the high frequency of introns in *C. gattii* is the difference in the mitochondrial inheritance mechanism between these complexes. For *C. neoformans,* it is mostly uniparental, while in *C. gattii* it is frequently biparental [38]. This could favor the homing process by bringing together mitochondria populations with different patterns of autocatalytic intron presence, thus speeding up the spread of group I introns in the population.

The biparental mtDNA inheritance pattern in *C. gattii* may also be associated with the increased occurrence of multiple bands observed on PCR electrophoresis for a specific intron, a phenomenon that was more commonly observed in this species complex (Supplementary File S2). It is noteworthy that those bands corresponded to the expected range for the presence and absence of group I introns, suggesting that the fungal isolate contains distinct populations of mitochondria (heteroplasmy). The hypothesis of sample contamination in these cases was ruled out after consecutive colony isolations, followed by new DNA extractions and PCRs.

The association between biparental mitochondrial inheritance and the homing process (intron invasion) has already been experimentally demonstrated by knocking out the transcription factor *sxi1*α, which is responsible for uniparental mitochondrial inheritance in *C. neoformans* [39]. In typical crosses between MATa and MATα strains, the progeny almost exclusively inherit their mitogenome from the MATa parent. However, by disrupting *sxi1*α, Yan et al. [40] observed the presence of introns with homing endonucleases from the MATα parent in the majority (>95%) of the progeny. This suggests that the *sxi1*α transcription factor inhibits the dissemination of introns containing HEGs in the mitochondrial genome of *C. neoformans* by determining uniparental mitochondrial inheritance. Indeed, it has been proposed in the literature that throughout the evolution of the Eukarya Domain, the pattern of mtDNA inheritance from one parental cell only may have been selected in response to the potential harmful spread of introns in the mitogenome [41,42,43].

The size polymorphism of the intronic sequences observed in Supplementary File S2, for the *cob* and *cox1* genes, can be explained by the presence or absence of group I introns, as well as the presence of homing endonuclease genes (HEGs) within these elements. HEGs were identified in 29 out of the 36 sequenced introns, all belonging to the LAGLIDADG family, according to CDD-NCBI. Once functional, these endonucleases contribute to the dispersion of their introns, leading to a super-Mendelian inheritance pattern in heterozygous crosses or, in some cases, they may also function as maturases, enzymes that stabilize the secondary structure of introns, assisting their auto-splicing [44,45,46]. Although the conserved domain of HEG was identified in the introns studied here, only experimental approaches can prove whether they are functional in recognizing an empty insertion site or are functioning as maturases as well.

The group I introns sequenced in this study were classified into the IA and ID families for the *cob* gene, and the IA and IB families for the *cox1* gene. Notably, group I introns occupying the same insertion site from different fungal isolates were consistently identified as belonging to the same family, suggesting a single origin for all of them, possibly through ancestral invasion. To investigate this, individual phylogenetic analyses were conducted for the introns in the *cob* and *cox1* genes (Figure 6 and Figure 7, respectively), followed by a combined analysis with the intronic *mtLSU* sequences (Figure 8). The phylogeny revealed that group I introns from the same position formed monophyletic groups, ruling out the possibility of a recent heterologous invasion. However, the close proximity of introns 2 and 3, as well as introns 4 and 5 in *cox1*, suggests that an intron from one site may have subsequently invaded other sites in the same gene in the past.

A new group I intron, not described in the reference sequences, was found occupying a different position in the FC9 isolate (VGIV), between the second and third intronic insertion sites of *cox1* (*cox1* 2–3). A BLAST search revealed that this intron is similar to introns from other fungi, such as to the fourth intron of the *cox1* gene from *Arthonia susa*, an ascomycete-lichen, and from *Phellinotus piptadeniae*, a basidiomycete wood-decay fungi, and also to a nuclear sequence from *Apiotrichum mycotoxinovorans*, an opportunistic basidiomycete yeast. This strongly suggests a horizontal transfer of this intron and explains why this intron was placed as an external group in the phylogeny, even though it continued to group with the *cox1* introns. In fact, group I introns laterally transferred tend to invade homologous sites in the new host, so it is most probable that this intron 2–3 has come from the *cox1* gene from other species [16]. Studies indicate that ecological proximity may be the key factor for horizontal gene transfer (HGT) [47]. This is probably the case of the HGT evidenced here, since *C. gattii* species are frequently found on trees, sharing the same habitat with other environmental fungi, such as the species mentioned above. The fact that the greatest similarities and lower e-values were observed between this intron and introns from non-*Cryptococcus* fungi provides strong evidence for horizontal transfer. However, since there are numerous non-sequenced environmental fungi, it is not possible to precisely determine from which species this intron originated. We can only affirm that it did not come from another *Cryptococcus* species. Considering the occurrence of such HGT, it is more parsimonious to hypothesize that the position occupied by this intron is a more recent insertion site, as it was absent in all other *Cryptococcus* genotypes, including another isolate that also belongs to the VGIV genotype. However, in this case, a recent loss could also explain why one VGIV isolate has it and the other does not. Despite being a recent invasion, it can be observed that the branch of this intron is more basal in relation to the other *cox1* introns. This can be explained by the fact that it originated from a distant lineage species rather than another *Cryptococcus* fungus.

The phylogenetic analysis was used to uncover similarity relationships among introns from different sites. In the case of *cob* introns, it can be observed that the group I introns from positions 1 and 2 were more closely related. Overall, the *cob* and *cox1* introns form monophyletic groups, although with the exception of the case discussed above, which confirms that introns from the same gene have more similarities with each other than with introns from other genes. In other words, it is highly likely that an intron has invaded multiple sites within the same gene in the past, prior to the divergence of *Cryptococcus* species, so that all introns of the same gene share a common ancestor. Moreover, it is noteworthy that in the cladograms presented in Figure 6 and Figure 7, the intronic sequences of *cob* and *cox1* from different *Cryptococcus* isolates lack informative sites to differentiate among cryptic species. Notably, even the complexes cannot be distinguished, as some introns from *C. neoformans* are more closely related to certain introns from *C. gattii* than to other introns from *C. neoformans*.

The addition of *mtLSU* intronic sequences to our phylogeny (Figure 8) made it more evident that the introns from *cob* and *cox1* form monophyletic groups, indicating that introns from the same gene are more similar to each other than to introns from other genes, but the same could not be observed for the *mtLSU* introns, which were shown to be paraphyletic, as previously observed by Gomes et al. [25].

The phylogeny showed that the group I introns studied here are not good molecular markers for differentiating genotypes or cryptic species of *Cryptococcus* through gene sequencing. It was also noted that a single PCR reaction would not be able to differentiate these fungi through the band pattern observed in electrophoresis. However, it is noteworthy that once the species complex to which a particular isolate belongs is known (*C. neoformans* or *C. gattii*), two PCR reactions amplifying different intronic regions (Table 3) can distinguish the species, as described by Hagen et al. [6]. Prior differentiation of species complexes is necessary because some band patterns may be similar between species from different species complexes, such as *C. neoformans* and *C. tetragattii*. It should be noted that the PCR reactions suggested here were based on a preliminary analysis and were unable to differentiate between the VNI and VNII genotypes (belonging to the same species). Furthermore, additional tests are still necessary, including more isolates, particularly those from underrepresented genotypes in our fungal collection.

For the first time, a PCR-based method utilizing group I introns has been proposed to differentiate cryptic species in *Cryptococcus*. This approach is faster and more cost-effective compared to other techniques, such as PCR-RFLP or MLST, commonly used for this purpose, and has great potential for use in research laboratories. Additionally, the data obtained in this study regarding the distribution of group I introns in crucial mitochondrial genes in pathogenic *Cryptococcus* were at least intriguing, taking into account that *cob* encodes for cytochrome b, an essential component of the functional unit of Complex III of the electron transport chain, and *cox1* encodes for the subunit 1 of cytochrome c oxidase (Complex IV), which plays a role in the final step of the respiratory chain [48]. The expression of these genes depends on successful intron splicing, which means that the efficiency of intron autocatalysis may regulate the levels of *cob* and *cox1* products in mitochondria. The data obtained in this study regarding the abundance of group I introns in crucial mitochondrial genes in pathogenic *Cryptococcus* may serve as a starting point for investigating their impact on mitochondrial gene expression and, consequently, fungal fitness in parasitic conditions.

## Figures and Tables

**Table 1:** Primers for the amplification of group I introns present in *cob* and *cox1* genes from *Cryptococcus*.

Gene	Intron	Forward Primer (5′-3′)	Reverse Primer (5′-3′)
*cob*	1	*COBE1F* ATGTTAACTACGGATGGATG	*COB1E2R* GTACCAATCCAAGGAATAGC
	2	*COB1E2F* GCTATTCCTTGGATTGGTAC	*COB1E3R* CAGAAGGCAAATCGTGTTAG
*cox1*	1	*COX1E1F* CCAACTATACAATGTAATCGC	*COX1E2R* TGAAATCATACCRAAACCTGG
	2	*COX1E2F* CAGGTTTYGGTATGATTTCAC	*COX1E3R* GCTGCTGTAAAGTAAGCTCG
	3	*COX1E3F* CGAGCTTACTTTACAGCAGC	*COX1E4R* ATTGTAAATAGAGCTACAAATCC
	4 and 5	*COX1E4F* GGATTTGTAGCTCTATTTACAAT	*COX1E6R* GAGGCATTCCAGCTAGTCC

**Table 2:** Sequence analysis of group I introns present in *cob* and *cox1* mitochondrial genes from different isolates from *C. neoformans* and *C. gattii* complexes, regarding intron size, classification, occupation site, and presence of homing endonuclease conserved domains.

Sequenced Introns		Classification (RNAweasel)		Homing Endonuclease (Conserved Domain Database)	
Gene	Strain/Genotype	Family	e-Value	Family	HEG Accession/e-Value
*cob*	BT8/VG2 (1)	ID	8.02 × 10^−36^	–	–
	CFP61/VGIII (1)	ID	9.18 × 10^−38^	LAGLIDADG	Accession cl03916/E-value 4.32 × 10^−14^
	CFP56/VNII (1)	ID	4.29 × 10^−36^	LAGLIDADG	Accession pfam03161/E-value 6.51 × 10^−58^
	CFP58/VNIV (1)	ID	4.29 × 10^−36^	LAGLIDADG	Accession pfam03161/E-value 7.64 × 10^−56^
	FC4/VNIII (1)	ID	3.16 × 10^−36^	LAGLIDADG	Accession cl03916/E-value 5.31 × 10^−12^
	BT8/VG2 (2)	IA	1.49 × 10^−22^	LAGLIDADG	Accession cl24136/E-value 4.06 × 10^−3^
	CFP58/VNIV (2)	IA	2.06 × 10^−22^	LAGLIDADG	Accession cl24136/E-value 4.09 × 10^−3^
	BT26/VNI (2)	IA	3.10 × 10^−22^	LAGLIDADG	Accession cl24136/E-value 5.12 × 10^−3^
	CFP59/VGI (2)	IA	3.68 × 10^−20^	–	–
	HGT17/VNI (2)	IA	1.38 × 10^−18^	LAGLIDADG	Accession cl24136/E-value 5.13 × 10^−3^
*cox1*	FC4/VNIII (1)	IB	2.70 × 10^−25^	LAGLIDADG	Accession pfam00961/E-value 2.92 × 10^−6^
	BT28/VNIV (1)	IB	2.70 × 10^−25^	LAGLIDADG	Accession pfam00961/E-value 2.92 × 10^−6^
	BT27/VGII (1)	IB	1.41 × 10^−24^	LAGLIDADG	Accession cl08299/E-value 3.83 × 10^−7^
	FC3/VGIII (1)	IB	2.54 × 10^−25^	LAGLIDADG	Accession cl08299/E-value 2.92 × 10^−6^
	CFP56/VNII (2)	IB	1.48 × 10^−22^	LAGLIDADG	Accession pfam14528/E-value 4.26 × 10^−4^
	Fc4/VNIII (2)	IB	1.48 × 10^−22^	LAGLIDADG	Accession pfam14528/E-value 3.20 × 10^−4^
	BT28/VNIV (2)	IB	9.30 × 10^−23^	LAGLIDADG	Accession pfam14528/E-value 1.56 × 10^−4^
	BT27/VGII (2)	IB	8.97 × 10^−20^	LAGLIDADG	Accession pfam14528/E-value 2.75 × 10^−4^
	FC3/VGIII (2)	IB	8.65 × 10^−23^	LAGLIDADG	Accession pfam14528/E-value 1.55 × 10^−4^
	FC9/VGIV (2)	IB	3.61 × 10^−29^	LAGLIDADG LAGLIDADG	Accession pfam00961/E-value 1.67 × 10^−12^ Accession cl24136/E-value 9.98 × 10^−3^
	LCR2002368/VNI (3)	Similar to IB	1.52 × 10^−29^	LAGLIDADG	Accession cl03916/E-value 1.25 × 10^−28^
	FC4/VNIII (3)	Similar to IB	5.06 × 10^−29^	LAGLIDADG	Accession pfam03161/E-value 1.31 × 10^−49^
	BT28/VNIV (3)	Similar to IB	5.80 × 10^−30^	LAGLIDADG	Accession pfam03161/E-value 5.30 × 10^−53^
	CFP59/VGI (3)	Similar to IB	5.80 × 10^−30^	LAGLIDADG	Accession pfam03161/E-value 2.42 × 10^−51^
	CFP61/VGIII (3)	Similar to IB	5.68 × 10^−31^	–	–
	FC4/VNIII (4)	Similar to IB	1.12 × 10^−9^	LAGLIDADG	Accession pfam00961/E-value 4.68 × 10^−12^
	BT28/VNIV (4)	Similar to IB	1.30 × 10^−9^	LAGLIDADG	Accession pfam00961/E-value 4.68 × 10^−12^
	CFP59/VGI (4)	Similar to IB	1.30 × 10^−9^	LAGLIDADG LAGLIDADG	Accession pfam00961/E-value 5.67 × 10^−13^ Accession cl24136/E-value 8.57 × 10^−3^
	BT27/VGII (4)	Similar to IB	1.98 × 10^−7^	LAGLIDADG	Accession pfam00961/E-value 1.07 × 10^−11^
	CN894/VGII (4)	NI	NI	LAGLIDADG	Accession pfam00961/E-value 7.76 × 10^−12^
	FC3/VGIII (4)	NI	NI	LAGLIDADG	Accession pfam00961/E-value 4.80 × 10^−12^
	FC9/VGIV (4)	Similar to IB	1.37 × 10^−9^	LAGLIDADG LAGLIDADG	Accession pfam00961/E-value 5.59 × 10^−13^ Accession cl24136/E-value 8.49 × 10^−3^
	Fc4/VNIII (5)	IA	7.78 × 10^−17^	–	–
	BT28/VNIV (5)	IA	8.84 × 10^−17^	–	–
	CN894/VGII (5)	IA	2.98 × 10^−19^	–	–
	FC3/VGIII (5)	IA	9.06 × 10^−17^	–	–

The values in parentheses in the second column refer to the insertion site of the group I intron in the gene (1 and 2 in *cob*, 1, 2, 3, 4, and 5 in *cox1*). NI: not identified, (–): absence of HEG.

**Table 3:** Suggested PCRs for differentiation of genotypes/cryptic species after the definition of species complexes, *C. neoformans* or *C. gattii*.

**PCR Target**	**Primer’s Pair**	***C. neoformans* Complex Expected Product Sizes (bp)**			
		***C. neoformans***		***Hybrid***	***C. deneoformans***
		**VNI**	**VNII**	**VNIII**	**VNIV**
*mtLSU* *	*CryLSUF/CryLSUR*	300 or 1300	1300	2000 or 2500	300 or 1900
introns 4 and 5 of *cox1*	*COX1E4F/COX1E6R*	300	300	1600	1600
**PCR Target**	**Primer’s Pair**	***C. gattii* Complex Expected Product Sizes (bp)**			
		***C. gattii***	***C. deuterogattii***	***C. bacillosporus***	***C. tetragattii***
		**VGI**	**VGII**	**VGIII**	**VGIV**
*mtLSU* *	*CryLSUF/CryLSUR*	300 or 1100	300	1800	1300
intron 1 of *cob*	*COBE1F/COB1E2R*	1200	250 or 600	250 or 1200	250

* Primers described by Gomes et al. [25] flanking all introns from the gene *mtLSU*.

## Data Availability

Not applicable.

## Supplementary Materials

The following supporting information can be downloaded at: https://www.mdpi.com/article/10.3390/jof9060629/s1, File S1: *Cryptococcus neoformans* and *Cryptococcus gattii* isolates used in this work; File S2: PCR products obtained for *cob* and *cox1* introns from different *C. neoformans* and *C. gattii* isolates; File S3: Size variation of PCR products for group I introns in *cob* and *cox1* genes; File S4: Group I introns and homing endonuclease genes sequences (fasta).
