# Supplementary material for: Distribution and Polymorphisms of Group I Introns in Mitochondrial Genes from Cryptococcus neoformans and Cryptococcus gattii
Source: J Fungi (Basel). 2023 May 30;9(6):629. doi: 10.3390/jof9060629 (PMC10299519; doi:10.3390/jof9060629)
Supplement: Supplementary file 1 [file jof-09-00629-s001.zip › Supplementary File S1_Isolates.pdf]

Supplementary File S1 - Isolates

Table S1. *Cryptococcus neoformans and Cryptococcus gattii* isolates used in this work.

Genotype	Isolate ID	Isolate origin
VNI	BT1	HC-SP
	BT2	HC-SP
	BT3	HC-SP
	BT4	HC-SP
	BT5	HC-SP
	BT6	HC-SP
	BT7	HC-SP
	BT9	HC-SP
	BT10	HC-SP
	BT11	HC-SP
	BT12	HC-SP
	BT13	HC-SP
	BT15	HC-SP
	BT16	HC-SP
	BT18	NI
	BT22	HV-SP
	BT23	HV-SP
	BT25	HV-SP
	BT26	HV-SP
	HGT1	HGT-RN
	HGT3	HGT-RN
	HGT6	HGT-RN
	HGT7	HGT-RN
	HGT8	HGT-RN
	HGT9	HGT-RN
	HGT11	HGT-RN
	HGT12	HGT-RN
	HGT13	HGT-RN
	HGT15	HGT-RN
	HGT16	HGT-RN
	HGT17***	HGT-RN
	HGT18***	HGT-RN
	UFRN1**	EI-RN
	UFRN2**	EI-RN
	PI1543	UFPI-PI
	PI1560	UFPI-PI
	CN216	IMT-SP
	LCR2002237	LACEN-RN
	LCR2002368	LACEN-RN
	CFP55	UFRGS-RS
VNII	FC5 (WM626)*	WM
	HGT2	HGT-RN
	CFP56	UFRGS-RS
VNIII	FC4 (WM628)*	WM
	CN117	IMT-SP
	CFP57	UFRGS-RS
VNIV	BT28	ATCC28958
	BT29	ATCC28957
	FC2 (WM629)*	WM
	FC7	FIOCRUZ-RJ
	CFP58	UFRGS-RS
VGI	BT14	HC-SP
	CFP59	UFRGS-RS
VGII	BT8	HC-SP
	BT17	HC-SP
	BT20	HV-SP
	BT21	HV-SP

	BT24	HV-SP
	BT27	NI
	FC1 (WM178)*	WM
	FC6	FIOCRUZ-RJ
	HGT5	HGT-RN
	HGT10	HGT-RN
	HGT14	HGT-RN
	HSL1	HSL-RN
	PI1401	UFPI-PI
	CG606	IMT-SP
	CG201	IMT-SP
	CG751	IMT-SP
	CG769	IMT-SP
	CN894	IMT-SP
	CN508	IMT-SP
VGIII	FC3 (WM161)*	WM
	CFP61	UFRGS-RS
VGIV	FC9 (WM779)*	FIOCRUZ-RJ
	CFP62	UFRGS-RS

Legend: NI – isolates with uninformed origin (from mycological collection of IBB-UNESP, Botucatu, São Paulo State, Brazil); HC - Botucatu Clinic hospital/UNESP, Botucatu, São Paulo State, Brazil; HV - Botucatu Veterinary hospital/UNESP, Botucatu, São Paulo State, Brazil; HGT - Giselda Trigueiro Hospital, Natal, Rio Grande do Norte State, Brazil; UFPI - Federal University of Piauí, Piauí State, Brazil; IMT-SP - Institute of Tropical Medicine of São Paulo, São Paulo, São Paulo State, Brazil; LACEN-RN, Central Laboratory of Rio Grande do Norte, Natal, Rio Grande do Norte State, Brazil; UFRGS-RS - Federal University of Rio Grande do Sul, Porto Alegre, Rio Grande do Sul State, Brazil; FIOCRUZ-RJ – Fundação Oswaldo Cruz, Rio de Janeiro, Rio de Janeiro State, Brazil; HSL-RN - São Lucas Hospital, Natal, Rio Grande do Norte State, Brazil; *WM – from Wieland Meyer collection, University of Sydney, Australia [5]; ** EI- Environmental Isolate from pigeons feces, Alecrim, Natal, Rio Grande do Norte State, Brazil; *** Two new isolates not genotyped by Gomes et al. [25].
