# Supplementary material for: Distribution and Polymorphisms of Group I Introns in Mitochondrial Genes from Cryptococcus neoformans and Cryptococcus gattii
Source: J Fungi (Basel). 2023 May 30;9(6):629. doi: 10.3390/jof9060629 (PMC10299519; doi:10.3390/jof9060629)
Supplement: Supplementary file 1 [file jof-09-00629-s001.zip › Supplementary File S2_Amplicons.pdf]

Supplementary File S2 - Tables S1 and S2

Table S1. PCR products obtained for introns 1 and 2 of the *cob* gene from different *C. neoformans and C. gattii* isolates.

Genotype	Isolate	PCR intron 1 (bp)	PCR intron 2 (bp)
VNI	BT1	250	500
	BT2	250	1500
	BT3	250	1500
	BT4	250	1500
	BT5	250	1500
	BT6	250	1500
	BT7	250	1500
	BT9	250	1500
	BT10	250	1500
	BT11	250	500
	BT12	1000	1500
	BT13	250	1500
	BT15	250	1500
	BT16	250	1500
	BT18	250	1500
	BT22	250	500
	BT23	250	1500
	BT25	250	1500
	BT26	250	500
	HGT1	250	1500
	HGT3	250	500 and 1500
	HGT6	250	1500
	HGT7	250	1500
	HGT8	250	1500
	HGT9	250	1500
	HGT11	250	1500
	HGT12	250	1500
	HGT13	250	1500
	HGT15	250	1500
	HGT16	250	1500
	HGT17	250	1500
	HGT18	250	1500
	UFRN1	250	500
	UFRN2	250	500
	PI1543	250	1500
	PI1560	250	1500
	CN216	1200	1500
	LCR2002237	250	1500
	LCR2002368	1200	1500
	CFP55	250	1500
VNII	FC5 (WM626)	250	500
	HGT2	1200	500
	CFP56	1200	500
VNIII	FC4 (WM628)	1200	500
	CN117	1200	1500
	CFP57	1200	500
VNIV	BT28	1200	1500
	BT29	1200	1500
	FC2 (WM629)	1200	1500
	FC7	1200	1500
	CFP58	1200	1500
VGI	BT14	1200	500
	CFP59	1200	800
VGII	BT8	600	1500
	BT17	600	1500
	BT20	600	1500
	BT21	600	1500
	BT24	600	1500
	BT27	250	500
	FC1 (WM178)	600	1500
	FC6	250	1500
	HGT5	250	500
	HGT10	600	1500
	HGT14	600	1500
	HSL1	600	1500
	PI1401	600	1500
	CG606	600	1500
	CG201	600	1500
	CG751	250	500
	CG769	600	1500
	CN894	600	1500
	CN508	600	1500
VGIII	FC3 (WM161)	250	500
	CFP61	1200	500 and 1500
VGIV	FC9 (WM779)	250	500
	CFP62	250	500

In red the PCR products that were sequenced. Products of 250 and 500 bp, respectively for introns 1 and 2, indicate the absence of this element in these insertion sites, because they refer to the flanking exons only.

Table S2. PCR products obtained for introns 1, 2, 3 and 4 and 5 of the *cox1* gene from different *C. neoformans and C. gattii* isolates.

Genotype	Isolate	PCR intron 1 (bp)	PCR intron 2 (bp)	PCR intron 3 (bp)	PCR intron 4 e 5 (bp)
VNI	BT1	600	200	160	300
	BT2	600	200	160	300
	BT3	600	200	160	300
	BT4	600	200	160	300
	BT5	600	200	160	300
	BT6	600	200	160	300
	BT7	600	200	160	300
	BT9	600	200	160	300
	BT10	600	200	160	300
	BT11	600	200	160	300
	BT12	600	200	1200	300
	BT13	600	200	1200	300
	BT15	600	200	160	300
	BT16	600	200	160	300
	BT18	600	200	160	300
	BT22	600	200	160	300
	BT23	600	200	160	300
	BT25	600	200	160	300
	BT26	600	200	160	300
	HGT1	600	200	160	300
	HGT3	600	200	160	300
	HGT6	600	200	160	300
	HGT7	600	200	160	300
	HGT8	600	200	160	300
	HGT9	600	200	160	300
	HGT11	600	200	160	300
	HGT12	600	200	160	300
	HGT13	600	200	160	300
	HGT15	600	200	160	300
	HGT16	600	200	160	300
	HGT17	600	200	160	300
	HGT18	600	200	160	300
	UFRN1	600	200	160	300
	UFRN2	600	200	160	300
	PI1543	600	200	160	300
	PI1560	600	200	160	300
	CN216	600	200	1200	300
	LCR2002237	600	600	160	300
	LCR2002368	600	600	1200	300
	CFP55	600	600	160	300
VNII	FC5 (WM626)	1500	1200	160	300
	HGT2	1500	1200	160	300
	CFP56	1500 and 600	1200 and 200	160	300
VNIII	FC4 (WM628)	1500	1200	1200	1600 and 300
	CN117	1500	1200	1200	1600
	CFP57	1500	1200	1200	1600 and 300
VNIV	BT28	1500	1200 and 200	1200	1600 and 300
	BT29	1500	1200	1200	1600 and 300
	FC2 (WM629)	1500	1200 and 200	160	1600 and 300
	FC7	600	1200 and 200	1200 and 160	1600 and 300
	CFP58	1500	1200	160	1600 and 300
VGI	BT14	600	200	160	300
	CFP59	NR	200	500	1400
VGII	BT8	1500	200	1200 and 160	2500 and 500 and 300
	BT17	1500 and 600	200	1200 and 160	2500 and 500 and 300
	BT20	600	200	160	300
	BT21	1500 and 600	200	1200 and 160	1400 and 300
	BT24	1500 and 600	200	1200 and 160	2500 and 300
	BT27	1500 and 600	1200	1200 and 160	1400 and 300
	FC1 (WM178)	1500 and 600	200	NR	2500 and 500 and 300
	FC6	1500 and 600	200	1200 and 160	2500 and 1400
	HGT5	1500	200	1200 and 160	1400 and 300
	HGT10	600	1200	1200 and 160	1400 and 300
	HGT14	1500	200	NR	300
	HSL1	1500 and 600	200	1200 and 160	2500 and 300
	PI1401	1500 and 600	200	1200 and 160	1400 and 300
	CG606	1500	200	1200 and 160	2500 and 300
	CG201	1500	200	1200 and 160	2500 and 500 and 300
	CG751	1500	200	1200 and 160	1400 and 300
	CG769	1500	200	1200 and 160	1400 and 300
	CN894	1500	200	1200 and 160	2500 and 500 and 300
	CN508	1500	200	1200	2500 and 500 and 300
VGIII	FC3 (WM161)	1500	1200 and 200	160	1500 and 300
	CFP61	1500 and 600	200	400	300
VGIV	FC9 (WM779)	1500 and 600	1300 and 200	160	1400
	CFP62	600	1300	160	1400

NR: non-reactive. In red the PCR products that were sequenced. Products of 600, 200, 160 and 300 bp, respectively for introns 1, 2, 3, 4/5 indicate absence of these elements in these insertion sites.
