# Supplementary material for: Distribution and Polymorphisms of Group I Introns in Mitochondrial Genes from Cryptococcus neoformans and Cryptococcus gattii
Source: J Fungi (Basel). 2023 May 30;9(6):629. doi: 10.3390/jof9060629 (PMC10299519; doi:10.3390/jof9060629)
Supplement: Supplementary file 1 [file jof-09-00629-s001.zip › Supplemetary File S3_Eletrophoresis.pdf]

Supplementary File S3 - Size variation of PCR products for group I introns in *cob* and *cox1* genes.

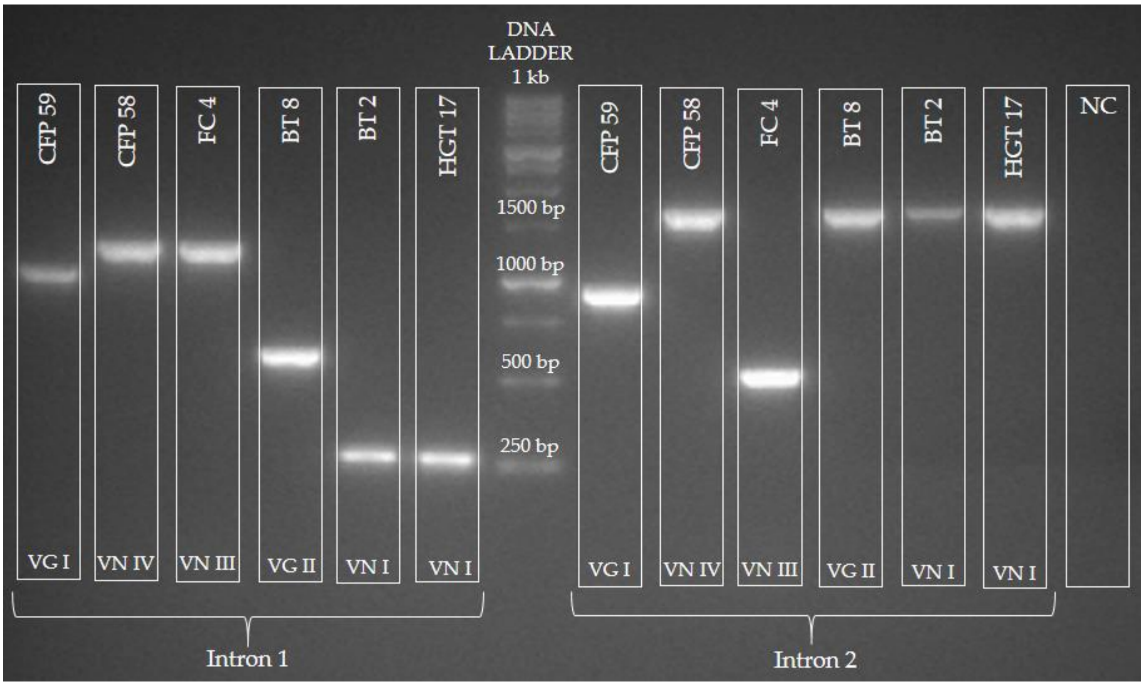

Figure S1. Size variation of PCR products for the introns from *cob* gene of isolates from *C. gattii* and *C. neoformans* species complexes. On the left side, the 250 bp bands indicate intron absence. On the right side, the 500 bp band indicates intron absence. NC: negative control.

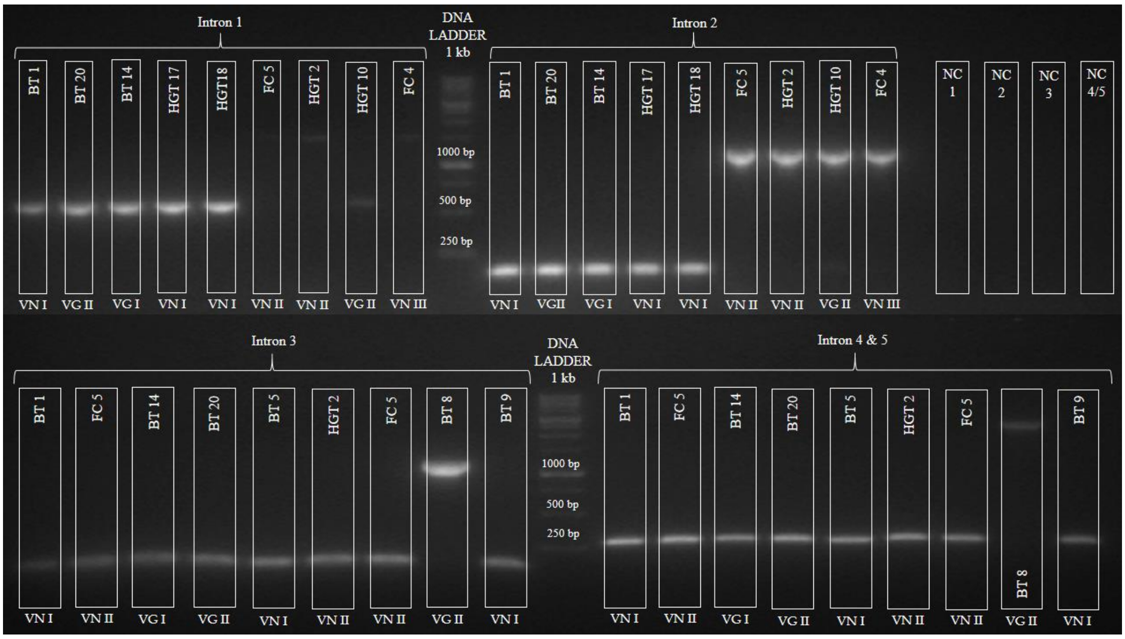

Figure S2. Size variation of PCR products for the introns from *cox1* gene of isolates from *C. gattii* and *C. neoformans* species complexes. The bands of approximately 600, 200, 160 and 300 bp of introns 1, 2, 3 and 4/5, respectively, represent the absence of introns. NC: negative control.
