# Supplementary material for: Distribution and Polymorphisms of Group I Introns in Mitochondrial Genes from Cryptococcus neoformans and Cryptococcus gattii
Source: J Fungi (Basel). 2023 May 30;9(6):629. doi: 10.3390/jof9060629 (PMC10299519; doi:10.3390/jof9060629)
Supplement: Supplementary file 1 [file jof-09-00629-s001.zip › Supplemetary File S4_Fasta sequences.pdf]

Supplementary File S4 - Group I intron sequences (red) highlighting homing endonuclease genes (blue)

>BT8_COB_INTRON1_VGII

ATTTAAAATTGCCCTATATGTTCTAATATTGTGATTTATACTGTTATAAGTATCAAATACTATCATATTATTATATCAC
AGTAAAAGAATATAGTCATATATTATATATGGCGACACTGATGAAAACGGTTAAAAATCTATAATTTCTGATTCAG
ACCGTCGGTTACTATTATAGTGGTCGCTACAGACTGGTTCATCGGTGGGTATCATCTAAAATGGTGCTTAATGTAC
AGTCGAAATTTCTATTAGATATAATATCTAATTACAAGGCCTGCATTTAAGACAGGTACATGGTCATATAATCGGA
AATTTTTTATCGTATATGATTAGATAATTTG

>BT8_COB_INTRON2_VGII

TTTATCCTTTGTCTAGTACCATTTCATGTATTTTTCAGTTCTGAATTTATACCTACTGTAGGTAATGTGAATAGCCGA
TCTATTCGTCCACGTAATAGTATGGACAAGAGTAAGTATCAAAATGTTAGCTATGACTTCATATCTATGTTTGCTGG
CTTTGTAGATGGAGACGGCTATATTAAGCTTACTCGGGCTACTGATGGTTATATCTCTTTTGAGCTAGTTATTTCTCT
AGACGTACGAGACGCAGAACTTCTACGCTATTTCTATTCTGTACTACAAGTAGGTCGTATTAACTATTACAATACT
ACAGTAAAGTATATTATTGGGCGAATCGATCTTCAAGAGATTGTATTTCCTCTTCTAATTCATCATGGTATTTATTTT
ATTACTCATACACATCGGCAACAATATGCACTAGCTCTACATGTACTAACTAATAATATTGTATGGTATGCTGACGT
ACCCACTAATCTTAATTCTAATATCTACCCTCTACCTACTACACCAGCTGGATATCTATCTATTCCTGGAATTGGGT
TGTTGGTTTTGTTATGGCTGAAGGATCATTCCACATTAAGGCTTCTAATGAGTTTTATTTTTCTATCCGACAACGTG
AACATATTACACTTTTTGAAGCTTTCCGAATTCTATTTAATACAACTCGAACTATTGACATATCTACTATTGGATATT
CAAAGTTTAATGTTTCTTCCGTTAAGGATCTAAATACTGTGGTTCAATTCTTTTCATTCAGGGCTACACCGGTTACA
AGAAGGTACAATATGACAATTGGATTAACAACATGCGAAACGTAAAGCGGTTTAAGAATATTAATCTGCCAGAA
CATAAAGGATATAAAGTTGCTTTTTGAGGTGACTCAAATTGAAAAAAAGGGTGAATTGCAAGAAACTCTTAATC
ATTTAACTTAACCCCTAGATTAAGACAATTTGCAGCTAAGTCTAGATTAGTATTTATATAAATCTAGAAAAGTTCA
ACGACTAGTTGGTGAGTAATACTAACAATAATCCAACCTAGAGCGCCCTTTCACTCTAAACGTAGTGAATGACAT
AGTCTAATCTTACTAGAAATAGTAAGCAGGCTTTTATAAGCTATAATGTTGTA

(HEG Accession cl24136, interval 148-366, E-value 1.30 × 10^{-03}

>CFP61_COB_INTRON1_VGIII

ATTTCAAATTGCCCGATATGTTCTAATACTGTGATTTATGCAGTTGTACTTGTACTTATTAATGTATATCTTCGGTCT
GGAATGCTAGCTAAGCGAATTTCAGCTATTAGCCGAGTAGGACCCCATAATATAACTATTCTTAGCATTATTACTG
GTTCTCTCCTTGGAGATGGTCATGCTGAACAGCGAGTTCAAGGAAATGGTACTCGTATTACTTTCTATCAAGCAG
AGAGTCATATAGAATATCTACTATGGCTGCATGAACTTATCTCTTCTCTTGGTTATTGTAGCCCTATCACACCTAAA
CACGTGTTGATAATGGAATCACTCGGTACGTAATTCGATTCCGAACTTACACATATAGTAGTTTTAACTTTATCTTT
GGTATGTTAACGGCATTAAGATTGTACCAGCTAATATTGCCATGTATCTTACTCCACTAGCACTTGCTATTTGGATT
ATGGATGATGGTGGTCGAATGGGTTACGGTCTAAAGCTAGCAACAAACTGCTTTACATATAATGACTGCGTATTT
CTTTCTAACACTCTAGCAGAACTATATGGAATTAAGTCTTCAGTACAAAATGCTGGGGTACCTAACCAGTATCATA
TTTATGTGTGGTCCGAATCTATGCCTCTACTACGAGATCTAGTTCGACCATATTTTGTACCATCTATGCTTTATAAGC
TAGGTGAATAACCCTTATATCATATAATATTCCCGATCACAGTATAAGAATATAGTCATATTTATATATGGCGACAC
TGATGAAAACGGTTAAAAATCTATAATTTCTGATTAAGACCGTCGGTTACTATAATATTATGGATATTATTAGTGGT
CGCTACAGACTGGTTCATCGGTGGGTATCATCTAAAATGATGCTTAATGTACAGTCGGAATTTCTATTAGATATATC
TAATTATAAGGCCTGCGTTCCTTTTAATTTAGGATTTTAGACAGGTACATGGTCATATTCGGAAATTTTTTATCGTAT
ATGATTAGATAATTTG

(HEG Accession cl03916, interval 139-294, E-value 1.14 × 10^{-13})

(HEG Accession cl03916, interval 377-643, E-value 5.80 × 10^{-27})

>CFP56_COB_INTRON1_VNII

ATTTCAAATTGCCCGACATGTTCTAATACTGTGATTTATGCAGTTGTACTTGTACTTATTAATGTATATCTTCGGTCT
GGAATGCTAGCTAAGCGAATTTCAGCTATTAGCCGAGTAGGACCCCATAATATAACTATTCTTAGCATTATTACTG
GTTCTCTACTTGGAGATGGTCATGCTGAACAGCGAGTTCAAGGAAATGGTACTCGTATTACTTTCTATCAAGCAG
GTAGTCATATAGAATATCTACTATGGCTACATGAACTTATCTCTTCTCTTGGTTATTGTAGCCCTATCACACCTGTTA
TCCAAACACGTGTTGATAATGGAATCACTCGGTACGTAATTCGATTCCGAACTTACACATATAGTAGTTTTAACTT

TATCTTTTCAGATTGGTATGTTAACGGCATTAAGATTGTACCAGCTAATATTGCCATGTATCTTACTCCACTAGCAC
TTGCTATTTGGATTATGGATGATGGTGGTCGAATGGGTTACGGTCTAAAGCTAGCAACAAACTGCTTTACATATAA
TGACTGCGTATTTCTTTCTAACACTCTAGCAGAACTATATGGAATTAAGTCTTCAGTACAAAATGCTGGGGTACCT
AACCAGTATCATATTTATGTGTGGTCCGAATCTATGCCTCTACTACGAGATCTAGTTCGACCATATTTTGTACCATC
TATGCTTTATAAGCTAGGTGAATAACCTTATATCATATAATACTCCCCATCACAGTATAAGAATATAGTCATATTTAT
ATATGGCGACACTGATGAAAACGGTTAAAAATCTATAATTTCTGATTCAGACCGTCGGTTACTATAATATTATGGA
TATTATTAGTGGTCGCTACAGACTGGTTCATCGGTGGGTATCATCTAAAATGGTGCTTAATGTACAGTCGGAATTT
CTATTAGATATATCTAATTATAAGGCCTGCGTTCCTTTTAATTTAGGATTTAAGACAGGTACATGGTCATATTCGGA
AATTTTTTATCGTATATGATTAGATAATTTG

(HEG Accession pfam03161, interval 139-657, E-value 2.19 × 10^{-57})

>CFP58_COB_INTRON1_VNIV

ATTTCAAATTGCCCGACATGTTCTAATACTGTGATTTATGCAGTTGTACTAGTACTTATTAATGTATATCTTCGGTCT
GGAATGCTAGCTAAGCGAATTTCAGCTATTAGCCGAGTAGGACCCCATAATATAACTATTCTTAGCATTATTACTG
GTTCTCTACTTGGAGATGGTCATGCTGAACAGCGAGTTCAAGGAAATGGTACTCGTATTACTTTCTATCAAGCAG
GTAGTCATATAGAATATCTACTATGGCTACATGAACTTATCTCTTCTCTTGGTTATTGTAGCCCTATCACACCTGTTA
TCCAAACACGTGTTGATAATGGAATCACTCGGTACGTAATTCGATTCCGAACTTACACATATAGTAGTTTTAACTT
TATCTTTTCAGATTGGTATGTTAACGGCATTAAGATTGTACCTATTGCCATGTATCTTACTCCACTAGCACTTGCTAT
TTGGATTATGGATGATGGTGGTCGAATGGGTTACGGTCTAAAGCTAGCAACAAACTGCTTTACATATAATGACTG
CGTATTTCTTTCTAACACTCTAGCAGAACTATATGGAATTAAGTCTTCAGTACAAAATGCTGGGGTACCTAACCAG
TATCATATTTATGTGTGGTCCGAATCTATGCCTCTACTACGAGATCTAGTTCGACCATATTTTGTACCATCTATGCTT
TATAAGCTAGGTGAATAACCTTATATAATACTCCCCATCACAGTATAAGAATATAGTCATATTTATATATGGCGACA
CTGATGAAAACGGTTAAAAATCTATAATTTCTGATTCAGACCGTCGGTTACTATAATATTATGGATATTATTAGTGG
TCGCTACAGACTGGTTCATCGGTGGGTATCATCTAAAATGGTGCTTAATGTACAGTCGGAATTTCTATTAGATATAT
CTAATTATAAGGCCTGCGTTCCTTTTAATTTAGGATTTAAGACAGGTACATGGTCATATTCGGAAATTTTTTATCGT
ATATGATTAGAGAATTTG

(HEG Accession pfam03161, interval 139-651, E-value 3.54 × 10^{-55})

>CFP58_COB_INTRON2_VNIV

TTTATCCTTTGTCTAGTATCATTTCATGTATTTTTCAGTTCAGAATTTCTACCTACTGTAGGTAATATGAATAGTCGAT
CTATTCATCCACGTAATAGTAAGGACAAGAGTAAGTATCAAAATGTTAGCTATGACTTCATATCTATGTTTGCTGG
CTTTGTAGATGGAGACGGCTATATTAAGCTTACTCGGGCTACTGATGGTTATATCTCTTTTGAGCTAGTTATTTCTCT
AGACGTACGAGACGCAGAACTTCTACGCTATTTCTATTCTGTACTACAAGTAGGTCGTATTAACTATTACAATACT
ACAGTAAAGTATATTATTGGGCGAATCGATCTTCAAGAGATTGTATTTCCTCTTCTAATTCATCATGGTATTTATTTT
CTTACTGATACACGTCGGCAACAATATGCACTAGCTCTACATGTACTAACTAATAATATTGTATGGTATGCAGACG
TACCCACTAATTTTAATTCTAATATCTACCCTCTACCTACTACTCCAGCTGGATATCTATCTATTCCTTTCTTTCGGA
ATTGGGTTGTTGGTTTTGTTATGGCTGAAGGATCATTCCACATTAAGGCTTCTAATGAGTTTTATTTCTCTATCCGA
CAACGTGAACATATTACACTTTTTGAAGCTTTCCGAATTCTATTTAATACGACTCGAACTATGGACATATCTACTAT
TGGATATTCAAAGTTTAATGTTTCTTCCGTTAAGGATCTAAATACTGTGGTTCAATTCTTTTCTCATTCCGGGCTAC
ATCCAATTGTAGGTTACAAGAAGGTACAATATGACAATTGGATTAACAACATACGAAACGTAAAGCGGTTTAAG
AATATTAATCTGCCAGAACATAAAGGATATAAAGTTGCTTTTTGAGGTGACTCAAATTGAAAAAAAAGGGTGAA
TTGCAAGAAACTCTTAATCATATAACTCAACCCCTAGATTAAGACAATTTGCAGCTAAGTCTAGATTAGTATTTAT
ATAAATCTAGAAAAGTTCAACGACTAGTTGGTGAGTAATACTAACAATAATCCAACCAAGAGCGCCCTTTCACT
CTAAACGTAGTGAATGACATAGTCTAATCTTACTAGAAATAGTAAGCAGGCTTTTATAAGCTATAATGTTGTA

(HEG Accession cl24136, interval 148-366, E-value 1.31 × 10^{-03})

> FC4_COB_INTRON1_VNIII

ATTTCAAATTGCCCGACATGTTCTAATACTGTGATTTATGCAGTTGTACTTGTACTTATTAATGTATATCTTCGGTCT
GGAATGCTAGCTAAGCGAATTTCAGCTATTAGCCGAGTAGGACCCCATAATATAACTATTCTTAGCATTATTACTG
GTTCTCTACTTGGAGATGGTCATGCTGAACAGCGAGTTCAAGGAAATGGTACTCGTATTACTTTCTATCAAGCAG
GTAGTCATATAGAATATCTACTATGGCTACATGAACTTATCTCTTCTCATGTAGCCCTATCACACCTGTTATCCAAA
CACGTGTTGATAATGGAATCACTCGGTACGTAATTCGATTCCGAACTTACACATATAGTAGTTTTAACTTTATCTTT
TCAGATTGGTATGTTAACGGCATTAAGATTGTACCTATTGCCATGTCTCCACTAGCACTTGCTATTTGGATTATGGA
TGATGGTGGTCGAATGGGTTACGGTCTAAAGCTAGCAACAAACTGCTTTACATATAATGACTGCGTATTTCTTTCT
AACACTCTAGCAGAACTATATGGAATTAAGTCTTCAGTACAAAATGCTGGGGTACCTAACCAGTATCATATTTATG

TGTGGTCCGAATCTATGCCTCTACTACGAGATCTAGTTCGACCATATTTTGTACCATCTATGCTTTATAAGCTAGGT
GAAGAACCTTATATAATACTCCCCATCACAGTATAAGAATATAGTCATATTTATATATGGCGACACTGATGAAAAC
GGTTAAAAATCTATAATTTCTGATTCAGACCGTCGGTTACTATAATATTATGGATATTATTAGTGGTCGCTACAGAC
TGGTTCATCGGTGGGTATCATCTAAAATGGTGCTTAATGTACAGTCGGAATTTCTATTAGATATATCTAATTATAAG
GCCTGCGTTCCTTTTAATTTAGGATTTAAGACAGGTACATGGTCATATTCGGAAATTTTTTATCGTATATGATTAGG
AGAATTG

(HEG Accession cl03916, interval 139-273, E-value 1.55×10^{-11})

(HEG Accession cl03916, interval 291-638, E-value 1.34×10^{-30})

>BT26_COB_INTRON2_VNI

TTTATCCTTTGTCTAGTATCATTTCATGTATTTTTCAGTTCAGAATTTCTACCTACTGTAGGTAATATGAATAGTCGAT
CTATTCATCCACGTAATAGTAAGGACAAGAGTAAGTATCAAAATGTTAGCTATGACTTCATATCTATGTTTGCTGG
CTTTGTAGATGGAGACGGCTATATTAAGCTTACTCGGGCTACTGATGGTTATATCTCTTTTGAGCTAGTTATTTCTCT
AGACGTACGAGACGCAGAACTTCTACGCTATTTCTATTCTGTACTACAAGTAGGTCGTATTAACTATTACAATACT
ACAGTAAAGTATATTATTGGGCGAATCGATCTTCAAGAGATTGTATTTCCTCTTCTAATTCATCATGGTATTTATTTT
CTTACTGATACACGTCGGCAACAATATGCACTAGCTCTACATGTACTAACTAATAATATTGTATGGTATGCAGACG
TACCCACTAATTTTAATTCTAATATCTACCCTCTACCTACTACTCCAGCTGGATATCTATCTATTCCTTTCTTTCGGA
ATTGGGTTGTTGGTTTTGTTATGGCTGAAGGATCATTCCACATTAAGGCTTCTAATGAGTTTTATTTCTCTATCCGA
CAACGTGAACATATTACACTTTTTGAAGCTTTCCGAATTCTATTTAATACGACTCGAACTATTGACATATCTACTAT
TGGATATTCAAAGTTTAATGTTTCTTCCGTTAAGGATCTAAATACTGTGGTTCAATTCTTTTCTCATTCAGGGCTAC
ATCCAATTGTAGGTTACAAGAAGGTACAATATGACAATTGGATTAACAATATACGAAACGTAAAGCGGTTTAAG
AATATTAATCTGCCAGAACATAAAGGATATAAAGTTGCTTTTTGAGGTGACTCAAATTGAAAAAAAGGGTGAATT
GCAAGAAACTCTTAATCATATAACTCAACCCCTAGATTAAGACAATTTGCAGCTAAGTCTAGATTAGTATTTATAT
AAATCTAGAAAAGTTCAACGACTAGTTGGTGAGTAATACTAACAATAATCCAACCTAGAGCGCCCTTTCACTCTA
AACGTAGTGAATGACATAGTCTAATCTTACTAGAAATAGTAAGCAGGCTTTTATAAGCTATAATGTTGTA

(HEG Accession cl24136, interval 148-366, E-value 1.62×10^{-03})

>CFP59_COB_INTRON2_ VGI

TTTATCCTTTGTCTAGTACCATTCCATGTATTTTTAAGTTCATAATTTCTACCTTCGAAGAGTAAATATTTAGACGTA
CGAGACGCAGAACTTCTACGTATTCAATACTACAAGTATTACAATACAACACATAAAGGATATAAAGTTGCTTTT
TGAGGTGACTCAAATTGAAAAAAAGGGTGAATTGCAAGAAACTCTTAATCATTTAACTCAACCCCTAGATTAAG
ACAATTTGCAGCTAAGTCTAGATTAGTATATATATAAATCTAGAAAAGTTCAACGACTAGTTGGTGAGTAATACTA
ACACTAATCCAACGTAGAGCGCCCTTTCACTATAAACGTAGCGAATGACATAGTCTAATCTTACTAGAAATAGTA
AGCAGGCTTTTATAAGATATAATGTAGTA

>HGT17_COB_INTRON2_VNI

TTTATCCTTTGTCTAGTATCATTTCATGTATTTTTCAGTTCAGAATTTCTACCTACTGTAGGTAATATGAATAGTCGAT
CTATTCATCCACGTAATAGTAAGGACAAGAGTAAGTATCAAAATGTTAGCTATGACTTCATATCTATGTTTGCTGG
CTTTGTAGATGGAGACGGCTATATTAAGCTTACTCGGGCTACTGATGGTTATATCTCTTTTGAGCTAGTTATTTCTCT
AGACGTACGAGACGCAGAACTTCTACGCTATTTCTATTCTGTACTACAAGTAGGTCGTATTAACTATTACAATACT
ACAGTAAAGTATATTATTGGGCGAATCGATCTTCAAGAGATTGTATTTCCTCTTCTAATTCATCATGGTATTTATTTT
CTTACTGATACACGTCGGCAACAATATGCACTAGCTCTACATGTACTAACTAATAATATTGTATGGTATGCAGACG
TACCCACTAATTTTAATTCTAATATCTACCCTCTACCTACTACTCCAGCTGGATATCTATCTATTCCTTTCTTTCGGA
ATTGGGTTGTTGGTTTTGTTATGGCTGAAGGATCATTCCACATTAAGGCTTCTAATGAGTTTTATTTCTCTATCCGA
CAACGTGAACATATTACACTTTTTGAAGCTTTCCGAATTCTATTTAATACGACTCGAACTATTGACATATCTACTAT
TGGATATTCAAAGTTTAATGTTTCTTCCGTTAAGGATCTAAATACTGTGGTTCAATTCTTTTCTCATTCAGGGCTAC
ATCCAATTGTAGGTTACAAGAAGGTACAATATGACAATTGGATTAACAATATACGAAACGTAAAGCGGTTTAAG
AATATTAATCTGCCAGAACATAAAGGATATAAAGTTGCTTTTTGAGGTGACTCAAATTGAAAAAAAGGGTGAATT
GCAAGAAACTCTTAAATCATATAACTCAACCCCTAGATTAAGACAATTTGCAGCTAAGTCTAGAATAAGTATTTA
TATAAATTCTAGAAAAGTTCAACGAATAAGTTGGTGAATAATACTAACAATAATCCAACCTAGAGCGCCCTTTCA
CTCTAAACGTAGTGAATGACATAGTCTAATCTTACTAGAAATAGTAAGCAGGCTTTTATAAGCTATAATGTTGTA

(HEG Accession cl24136, interval 148-366, E-value 1.63×10^{-03})

>FC4_COX1_INTRON1_VNIII

TTTAATTGGCCCCTTTGGAACGCGAGTTTCAATAAGCACTGTGCTATTTGCTGGAACAGTCTGCAACTAGGTGTC
ATTACTATGGTAATAAGTGGCCATCTATGGTCCCTATCTAGTCAAAATATGGCATCTAAGACTCAATCAGCAGGTA
ACCAACGACGCTATAATAGCAGTCTAGTAGGAACCTCAGAGACTACACGCACAGCTGCTCCTTTTTCACAAGCT
TTCTGCCAATGGCTAGCCGGTCTAATTGATGGTGATGGCTGTCTTCTCGTATCTAAGCAAGGGTACACTAGTCTAG
AAATCACAGTAGGGCTAGAGGATCTTCCTCTATTCCGATATATTCAAAATAAGCTAGGTGGCTCTATTAAGATGCG
AAGTGGGGCAAAGGCTTATCGCTATCGACTACACAACCAACAAGGAATGATTCAACTTGTAACTTGTATCAATG
GATACATCCGACATAGTACTCGTCTTCTACAACTACATCACGTTTGCCAACAACTTAACATTCCTATTATTACACC
AGGACCTCTATCTATCACATCTGCCTGGTTTGCTGGTTTCTTTGATGCAGATGGAACTATTGGGTTCTCTATTAAGA
ATGGTACTCCACAACTTAGTGTACGAGTCACAAATAAGCTTCTTCAAGATGTTTCATGTTATATGGATATCTTTGGT
GGAAATATTTACTTTGATAGTGCACAGAACGGTTATTATCAATGGTCTATTCAAAATAAGACTGATGTTATTCGTT
GTTCTAAGTATTTTATTCAATATTGTCGAAGCAATAAGTCTCGACGATTTTTCCTTATTGAAGAATATTACTCTCTTG
TGTCACTTCGGGCACATAAGTCTGAAAGTGATCATCATAAGGCATGGCTAGCCTTTATAGAAAAGTGGAATAAG
AAGTAGAAGATATAGTCCAAATAATATATTATAATATATTATAG

(HEG Accession pfam00961, interval 238-480, E-value 2.92 × 10^{-06})

>BT28_COX1_INTRON1_VNIV

TTTAATTGGCCCCTTTGGAACGCGAGTTTCAATAAGCACTGTGCTATTTGCTGGAACAGTCTGCAACTAGGTGTC
ATTACTATGGTAATAAGTGGCCATCTATGGTCCCTATCTAGTCAAAATATGGCATCTAAGACTCAATCAGCAGGTA
ACCAACGACGCTATAATAGCAGTCTAGTAGGAACCTCAGAGACTACACGCACAGCTGCTCCTTTTTCACAAGCT
TTCTGCCAATGGCTAGCCGGTCTAATTGATGGTGATGGCTGTCTTCTCGTATCTAAGCAAGGGTACACTAGTCTTG
AAATCACAGTAGGGCTAGAGGATCTTCCTCTATTCCGATATATTCAAAATAAGCTAGGTGGCTCTATTAAGATGCG
AAGTGGGGCAAAGGCTTATCGCTATCGACTACACAACCAACAAGGAATGATTCAACTTGTAACTTGTATCAATG
GATACATCCGACATAGTACTCGTCTTCTACAACTACATCACGTTTGCCAACAACTTAACATTCCTATTATTACACC
AGGACCTCTATCTATCACATCTGCCTGGTTTGCTGGTTTCTTTGATGCAGATGGAACTATTGGGTTCTCTATTAAGA
ATGGTACTCCACAACTTAGTGTACGAGTCACAAATAAGCTTCTTCAAGATGTTTCATGTTATATGGATATCTTTGGT
GGAAATATTTACTTTGATAGTGCACAGAACGGTTATTATCAATGGTCTATTCAAAATAAGACTGATGTTATTCGTT
GTTCTAAGTATTTTATTCAATATTGTCGAAGCAATAAGTCTCGACGATTTTTCCTTATTGAAGAATATTACTCTCTTG
TGTCACTTCGGGCACATAAGTCTGAAAGTGATCATCATAAGGCATGGCTAGCCTTTATAGAAAAGTGGAATAAG
AAGTAGAAGATATAGTCCAAATAATATATTATAATATATTATAG

(HEG Accession pfam00961, interval 238-480, E-value 2.92 × 10^{-06})

>BT27_COX1_INTRON1_VGII

TTTAATTGGCCCTTTTGGAATGTAAGTTCCAATAAGCACTGTGCTATTTGCTGGAACAGTCTGCAACTAGGTGTCA
TTACTATGGTAATAAGTGGCCATCAATGGTCCCTATCTAGTCAAAATATGACATCTAAGACTCAATCAGCAGGTA
ACCAACGACGCTATAATAGCAGTCTAGTAGGAACCTCAGAGACTACACGCACAGCTGCTCCTTTTTCACAAGCT
TTCTGTGAATGGCTAGCTGGTCTAATTGATGGTGATGGTTGTCTTCTCGTATCTAAGCAAGGGTACACTAGTCTTG
AAATCACTGTAGGGCTAGAGGATCTTCCTCTACTCCGATATATTCAAAACAAGCTAGGTGGTTCTATTAAGATGC
GAAGTGGAGCAAAGGCTTATCGCTATCGACTACACAACCGACAGGGAATAATTCAACTTGTAACTTGTATCAAT
GGATATATTCGACATAGTACTCGTCTTCTACAACTACATCATGTTTGCCAACAACTTAACATTTCTATTATTACACC
AGGATCTCTATCTGTTGCATCTGCCTGGTTTGCTGGTTTCTTTGATGCAGATGGTACTATAGGGTTCTCTATTAAGA
ATGGTATTCCACAACTTAGTGTACGAGTCACAAATAAGCTTCTTCAAGATGTTTCATGTTATATGGATATCTTTGGT
GGTAATATTTACTTTGATAGTGCACAGAACGGTTATTATCAATGGTCTATTCAAAGTAAGACTGATGTTATTCGTTG
TTCTAAGTATTTTGTTCAATATTGTCGAAGCAATAAGTCTCGACGATTTTTCCTTGTTGAAGAATATTATTCTCTAAT
TTCACTTAAGGCATATATGTCTGATAGTGATTATTATAAGGTATGGCTAGCCTTTATAGAAAAGTGGAATAAGAAG
TAGATGATATAGTCCAAATAATATATAATAATATATTATAG

(HEG Accession pfam00961, interval 238-480, E-value 3.83 × 10^{-07})

>FC3_COX1_INTRON1_VGIII

TTTAATTGGCCCCTTTGGAACGCGAGTTTCAATAAGCACTGTGCTATTTGCTGGAACAGTCTGCAACTAGGTGTC
ATTACTATGGTAATAAGTGGCCATCTATGGTCCCTATCTAGTCAAAATATGGCATCTAAGACTCAATCAGCAGGTA
ACCAACGACGCTATAATAGCAGTCTAGTAGGAACCTCAGAGACTACACGCACAGCTGCTCCTTTTTCACAAGCT
TTCTGCCAATGGCTAGCCGGTCTAATTGATGGTGATGGCTGTCTTCTCGTATCTAAGCAAGGGTACACTAGTCTTG
AAATCACAGTAGGGCTAGAGGATCTTCCTCTATTCCGATATATTCAAAATAAGCTAGGTGGCTCTATTAAGATGCG
AAGTGGGGCAAAGGCTTATCGCTATCGACTACACAACCAACAAGGAATGATTCAACTTGTAACTTGTATCAATG

GATACATCCGACATAGTACTCGTCTTCTACAACTACATCACGTTTGCCAACAACTTAACATTCCTATTATTACACC
AGGACCTCTATCTATCACATCTGCCTGGTTTGCTGGTTTCTTTGATGCAGATGGAACTATTGGGTTCTCTATTAAGA
ATGGTACTCCACAACTTAGTGTACGAGTCACAAATAAGCTTCTTCAAGATGTTTCATGTTATATGGATATCTTTGGT
GGAAATATTTACTTTGATAGTGCACAGAACGGTTATTATCAATGGTCTATTCAAAATAAGACTGATGTTATTCGTT
GTTCTAAGTATTTTATTCAATATTGTCGAAGCAATAAGTCTCGACGATTTTTCCTTATTGAAGAATATTACTCTCTTG
TGTCACTTCGGGCACATAAGTCTGAAAGTGATCATCATAAGGCATGGCTAGCCTTTATAGAAAAGTGGAATAAG
AAGTAGAAGATATAGTCCAAATAATATATTATAATATATTATAG

(HEG Accession pfam00961, interval 238-480, E-value 2.92 × 10^{-06})

>CFP56_COX1_INTRON2_VNII

TGCTACATAAATAAGCGAAAGCTTAATTATGTAACAGGTGGCCCTTACTGTTCTTCTATCTCAGAAGACAGTTATG
CAACATACTATATGCGGGAACGGCAGAATATTCTGATTTGTACTCGGATTACACTAATGACTATTGTGTATTTTTCG
ACAATAGCATATCCAGTAATAATCAATTCAGAACTGCCCAATCCGCAGGAAACCAACGCACTTTATTACATACTA
GGTCTTCTAGTAGAATATAGCATGCTAGTAGGATCCTCAGAGACTGTACGTATGTTCTCAACTATTATGACATCAC
ACGAGAAGTCAGATCTAAAGATTCGACAGTGGATTGCCGGAGTTATCGATGGCGACGGTTATTTTGGCATTAGTA
AGCTAGGTCATGTTTCACTAGAAGTCGTGATGGAACTACGTGATGAAGCTTGTCTATACAAGCTAAAGCATCGTT
ATGGCGGTTCTGTTAAGGCTACTTCGCATGCTAAGGCTCTTCGTTTCCGTCTACATCATAAGGCAGGGATTCTAGC
AGTTATTAATGATCTTAATGGTCTGCTATATAATCCTGTCCGTATCCAACAATTCCAAAAGGTATGCGCACTTTACA
ATGTGACATATCTAACACCTACACCACTAGAGTATATTAGTGCTTACATGTCAGGACTTTTTGACAGTGATGGTAG
CATCTATCTTAATATCACTTCTCAACAAGTATTTATTACTATCTCTCAAAAGTCACCTGAACTCCTTGATATTATCGC
TTCTGTTTATGGAGGAAAGGTATATTCATGTAACGCAAAGAATACTGCTTACAAGTGGACGGTATGTCGAAAGTC
GGAAGTACTAAGCCTAATTAATGACTATTTTCACTGGAACGGTTGCGTTTCAGCTAAGAACAAGCGTTTCGGAAT
AGTTAAGGAATTTTATCGACTATCATCTGTAGGTGCCACTAAGGCTACAATTGACTCACCATCAGGTAAGTCTCTC
CTACAATTTAAGGAACGTTGGGATAGCTACGACAACTCATAATAATTGTTAAAGATACAGTCCACTTATTTTTTAA
AAATAATAG

(HEG Accession pfam14528, interval 637-768, E-value 2.27 × 10^{-04})

>FC4_COX1_INTRON2_VNIII

TGCTACATAAATAAGCGAAAGCTTAATTATGTAACAGGTGGCCCTTACTGTTCTTCTATCTCAGAAGACAGTTACG
CAACATACTATATGCGGGAACGGCAGAATATTCTGATTTGTACTCGGATTACACTAATGACTATTGTGTATTTTTCG
ACAATAGCATATCCAGTAATAATCAATTCAGAACTGCCCAATCCGCAGGAAACCAACGCACTTTATTACATACTA
GGTCTTCTAGTAGAATATAGCATGCTAGTAGGATCCTCAGAGACTGTACGTATGTTCTCAACTATTATGACATCAC
ACGAGAAGTCAGATCTAAAGATCCGACAGTGGATTGCCGGAGTTATCGATGGCGACGGTTATTTTGGCATTAGTA
AGCTAGGTCATGTTTCACTAGAAGTCGTGATGGAACTACGTGATGAAGCTTGTCTATACAAGCTAAAGCATCGTT
ATGGCGGTTCTGTTAAGGCTACTTCGCATGCTAAGGCTCTTCGTTTCCGTCTACATCATAAGGCAGGGATTCTAGC
AGTTATTAATGATCTTAATGGTCTGCTATATAATCCTGTCCGTATCCAACAATTCCAAAAGGTATGCGCACTTTACA
ATGTGACATATCTAACACCTTCACCACTAGAGTATATTAGTGCTTACATGTCAGGACTTTTTGACAGTGATGGTAG
CATCAATCTTAATATCACTTCTCAACAAGTATTTATTACTATCTCTCAAAAGTCACCTGAACTCCTTGATATTATCG
CTTCTGTTTATGGAGGAAAGGTATATTCATGTAACGCAAAGAATACTGCTTACAAGTGGACGGTATGTCGAAAGT
CGGAAGTACTAAGCCTAATTAATGACTATTTTCACTGGAACGGTTGCGTTTCAGCTAAGAACAAGCGTTTCGGAA
TAGTTAAGGAATTTTATCGACTATCATCTGTAGGTGCCACTAAGGCTACAATTGACTCACCATCAGGTAAGTCTCT
CCTACAATTTAAGGAACGTTGGGATAGCTACGACAACTCATAATAATTGTTAAAGATACAGTCCACTTATTTTTTT
AAATAATAG

(HEG Accession pfam14528, interval 637-768, E-value 3.20 × 10^{-04})

>BT28_COX1_INTRON2_VNIV

TGCTACATAAATAAGCGAAAGCTTAATTATGTAACAGGTGGCCCTTACTGTTCTTCTATCTCAGAAGACAGTTACG
CAACATACTATATGCGGGAACGGCAGAATATTCTGATTTGTACTCGGATTACACTAATGACTATTGTGTATTTTTCG
ACAATAGCATATCCAGTAATAATCAATTCAGAACAGCCCAATCCGCAGGAAACCAACGCACTTTATTACATACTA
GGTCTTCTAGTAGAATATAGCATGCTAGTAGGATCCTCAGAGACTGTACGTATGTTCTCAACTATTATGACATCAC
ACGAGAAGTCAGATCTAAAGATCCGACAGTGGATTGCCGGAGTTATCGATGGCGACGGTTATTTTGGCATTAGTA
AGCTAGGTCATGTTTCACTAGAAGTCGTGATGGAACTACGTGATGAAGCTTGTCTATACAAGCTAAAGCATCGTT
ATGGCGGTTCTGTTAAGGCTACTTCGCATGCTAAGGCTCTTCGTTTCCGTCTACATCATAAGGCAGGGATTATAGC
AGTTATTAATGATCTTAATGGTCTGCTATATAATCCTGTCCGTATCCAACAATTCCAAAAGGTATGCGCACTTTACA
ATGTGACATATCTAACACCTTCACCACTAGAGTATATTAGTGCTTACATGTCAGGACTTTTTGACAGTGATGGTAG

CATCAATCTTAATATCACTTCTCAACAAGTATTTATTACTATCACTCAGAAGTCACCTGAACTCCTTGATATTATCG
CTTCTGTTTATGGAGGAAAGGTATATTCATGTAACGCAAAGAATACTGCTTACAAGTGGACGGTATGTCAAAAGT
CGGAAGTACTAAACCTAATTGATGACTATTTTCACTGGAACGGTTGCGTTTCAGCTAAGAACAAGCGTTTCGGAA
TAGTTAAGGAATTTTATCGACTATCATCTGTAGGTGCCACTAAGGCTACAATTGACTCACCATCAGGTAAGTCTCT
CCTACAATTTAAGGAACGTTGGGATAGCTACGACAACTCATAATAATTGTTAAAGATACAGTCCACTTATTTTTTT
AAATAATAG

(HEG Accession pfam14528, interval 637-768, E-value 1.56 × 10^{-04})

>BT27_COX1_INTRON2_VGII

TGCTACATAATAAGCGAAAGTCTTATTATGTAACAGGTGGCCCTTACTGTTCTCCTATCTCAGATAACAGTAACGC
AACATACTATATGCGGGAACGACAGATTATTCTGATTTGTACTCGGATTACACTAATGACTATTGTATATTATTCAA
TAATAGCATATCCAGTAATAATCAATTCAGAACTGTCCAATCCGCAGGAAACCAACGCACTTTATTACATACTAG
GTCTTCTAGTAGAATATAGCATGCTAGTAGGATCCTCAGAGACTGTACGTATGTTTTCAACTATTATGACATCACAT
GAGAAGTCGGATCTAAAGATTCGACAGTGGATTGCGGGAGTTATCGACGGAGACGGTTGTTTCTGCATTAGTAA
GCTAGGTTATGTTTCACTAGAAGTCGTGATAGAACCACGTGATGGAGCTTGTCTTTACAAGCTAAAGCATCGTTA
TGGCGGTTCTGTTAAGGCTACTTCACATGCTAAGGCTCTTCGTTTCCGTCTACATCATAAGGCAGGTATTCAAGCG
GTAATTAATGATCTTAATGGTCTGCTATATAATCCTGTCCGTATCCAACAATTCCAAAAGGTATGTACACTATATAA
TGTAACATATCTAACACCTTCACCACTAGAGTATATTAATGCTTACATGTCAGGACTTTTTGATAGTGATGGTAGC
ATCTATGTTAATATTATCTCTCAACAAGTATTTATTACTATCTCTCAAAAGTCACCTGAACTTCTTGATATTATCGCT
TCTGTTTATGGAGGAAAGGTATATCCATGTAATGCTAAGAAGACTGCTTACAAGTGGACGGTATGTCGAAAGTCT
GATGTACTAAATCTAATTAATGATTATTTTCACTGGAACGGTTGCGTTTCAGCTAAGAATAAGCGTTTTGGAATAG
TAAAGGAATTTTATCGACTATCATCTGAAGGTGCTACTAAGGCTACAATTGACTCACCATCAGGTAAGTCTCTTCT
ACAATTTAAGGAACGTTGGGATAGCTACGACAACTCATAATAGTCGTTAAAGATACAGTCCATTTATTTAAAATA
AAG

(HEG Accession pfam14528, interval 636-767, E-value 2.75 × 10^{-04})

>FC3_COX1_INTRON2_VGIII

TGCTACATAAATAAGCGAAAGCTTAATTATGTAACAGGTGGCCCTTACTGTTCTTCTATCTCAGAAGACAGTTACG
CAACATACTATATGCGGGAACGGCAGAATATTCTGATTTGTACTCGGATTACACTAATGACTATTGTGTATTTTTCG
ACAATAGCATATCCAGTAATAATCAATTCAGAACAGCCCAATCCGCAGGAAACCAACGCACTTTATTACATACTA
GGTCTTCTAGTAGAATATAGCATGCTAGTAGGATCCTCAGAGACTGTACGTATGTTCTCAACTATGATGACATCAC
ACGAGAAGTCAGATCTAAAGATCCGACAGTGGATTGCCGGAGTTATCGATGGCGACGGTTATTTTGGCATTAGTA
AGCTAGGTCATGTTTCACTAGAAGTCGTGATGGAACTACGTGATGAAGCTTGTCTATACAAGCTAAAGCATCGTT
ATGGCGGTTCTGTTAAGGCTACTTCGCATGCTAAGGCTCTTCGTTTCCGTCTACATCATAAGGCAGGGATTATAGC
AGTTATTAATGATCTTAATGGTCTGCTATATAATCCTGTCCGTATCCAACAATTCCAAAAGGTATGCGCACTTTACA
ATGTGACATATCTAACACCTTCACCACTAGAGTATATTAGTGCTTACATGTCAGGACTTTTTGACAGTGATGGTAG
CATCAATCTTAATATCACTTCTCAACAAGTATTTATTACTATCACTCAAAAGTCACCTGAACTCCTTGATATTATCG
CTTCTGTTTATGGAGGAAAGGTATATTCATGTAACGCAAAGAATACTGCTTACAAGTGGACGGTATGTCGAAAGT
CGGAAGTACTAAGCCTAATTAATGACTATTTTCACTGGAACGGTTGCGTTTCAGCTAAGAACAAGCGTTTCGGAA
TAGTTAAGGAATTTTATCGACTATCATCTGTAGGTGCCACTAAGGCTACAATTGACTCACCATCAGGTAAGTCTCT
CCTACAATTTAAGGAACGTTGGGATAGCTACGACAACTCATAATAATTGTTAAAGATACAGTCCACTTATTTTTTT
AAATAATAG

(HEG Accession pfam14528, interval 637-768, E-value 1.55 × 10^{-04})

>FC9_COX1_INTRON2-3_VGIV

TTTTAAACAAATTATAATTTGTGGTTGGCTCTCCTCTATCGCAAGATAAAGGTAATAAATTTCGCTATATGCTGGAA
CAGTTTAACGATAATCAATACTAATTTAAATATTAGTGAAAATTTGATTGTTTACACTCAATCAGCAGGTAACCAA
CAACTATATAGTTTAGTAGGAACCTCAGAGACTACACGCGAAAGACCTTATTATATTAGGTTAAGATATAGCCCG
ATCCTTCCTGTGAAGGAAGATAATTTTTTAACAAAATAAACATTTGCATTCTATTTATACTATCTATTTTGGTACTAC
TCTAGTTGACTATTCTGCTTCATATTATATGGCTTACTTTAGTAGTGGCTCTTTCACACTGTCAATGGTGATGATTCC
TATCATTCCCGGCCCTTCGTTTAATTTCACAGCATTTCAAACTAAGTTTGGTAAGGTTGTACCAGATACTTGGCTTC
AATGGTTTATTGGTTTTGCTGAAGGTGATGGTTCACTAGGAGTTTGGAATAAGCAGCTACGATTTATTCTAACTCA
AAAGGAAGGAAAGATCCTCTATGATATCCAATCTATATTTGGATTTGGAGAAGTTCTATATGTAGCACCAGGTATA
TATGGAAATAAGTATGGTTTTTATCGTTGGATTGTAACAAAGCCAGAAGATACATATCTTCTAATCCTTCTATTTAA
TGGAAATCTAGTTCTTCCTTATCGGATTCAACAACTAGGAGTCTGGCTCTCCACATATAATCTACGGAATACCGGT

GTATTCATAATTTCACTAATTACAACTGTTGTTACAATTACACTAAATGATGCGTGGCTATCTGGTTTTACTGATGC
TGAAGGATGTTTCAATGTTACTATTCGAAAGAACCGTCTTTATGGTCTAGTAATACGATTCCTTCTAGATCAAAAG
TGCCAAAAAACTCTAATGACTGTACAAACACTATTCAGTTTCGGTACAGTAGCACTACGAAATAACACTAATGG
AGTGTATCGATATGGAGCTAGTGGTATCGCCAATGTCTCAGTTATTCGCACTTACTTTGATAAGTTTCCTCTACGAA
CAAAGAAGCTATATTCTTACGAAAAGTGGTCACAAATTCACTCTATGGCACTTGATAATAAGCATCGATCTCAAG
AAGGATACGCCACTGTTATCGCTATTAAGGAGAGTATGAATGAATAATAAACTAATAAATATTTTGTTAAAAGAAT
TCCAACATG

(HEG Accession pfam00961, interval 824-1111, E-value 1.67×10^{-12})

(HEG Accession cl24136, interval 437-706, E-value 9.98×10^{-03})

>LCR2002368_COX1_INTRON3_VNI

AAACGAAGTGCTAGGCCCACTTCATATACTGGCCAACTATATTATTACTGTTTTCGATACTATCGCAGTACCTCTTA
GCTATCTATACCCTCGTGCAACCAAGTATAAGATTCTACCTAATAATACTTGTACTAGTATTGTACCTTATGGTACC
AATCAGGGTACCCTCTATTCAATATTATTACTCGAAATATGGTAGGTATCCCTTTCAATACTCTTTGTATTCTGGTTG
GCATGATCCTTTCTGATGCTTGGATGATGAAGTGGGGCAACTCTAATGCTCGACTAGCAATCAAGCAATCCATTC
GTAATTTTGGCTTTATCTGGCATTGTTTCCAGGTACTAGCTCATTATTGCTCAAGTATGCCCTCTATGGTACAATCG
ACTCTTCCTTCTGGTATTCACTATGGGGTAAGTGTTGTGACACGAACTCTACCTTGCTTTACTATTCTCTATCATAT
GTTCCATACTAATGGTGTGAAGACTATTCCTATGGATATCTTCGACTATCTTACTCCACCATGCCTTGCTTTTTGGA
TCATGGGAGATGTGGTGGAGGACTTTATCTACACACACAAACACTAGAAGAAAATATTCGACTACTTAATGCAC
TAACTATTAAGTATGATATTAAGGGGGTACTGGCGGAAGGACGTCATATTACAGTTCCCTCTATGCGGAAGCTATA
CCCTATTCTAGCGCCACATATGCATCCTTCTATGATGTACAAGATTTTTAAGAGTATCAAGTAATAATATAGCGGG
GCAAATTCGAGAGAACTCCTGTATATGGAATAAGTAACATATATAGGACAATCGTCGAACTAAATCATTAGTTGG
AAACGGCTAATGTAAAAGCGTAGAGACTATACGCCCCAGGTCAACCTAAGTATTTATTAATATACGGTGATCTAA
GACATAGTCCACTCCTTTAAGCAATTAAAGGTAGACTCTTTTAGCCTAGATCTGAAATGATCGAAGAAAGAGTAG
G

(HEG Accession cl03916, interval 223-549, E-value 1.25×10^{-28})

>FC4_COX1_INTRON3_VNIII

AAACGAAGTGCTAGGCCCACTTCATATACTGGCCAACTATATTATTACTGTTTTTAATATTAACACAGTACCTCTTA
ACTATCTGTACCCTCGTGCTACCAAGTATAAGATTCTACCTAATAATACTTGTACTAGTATTGTACCATATGGTACC
AATCTTGGTTCGACTGTAGGGTACCCTCAATTCAATATTATTATTCGAAATATGGTAGGTATCCCTTTCAATACTCT
TTGTATTCTGGTTGGCATGATCCTTTCTGATGCTTGGATGATGAAGCACAAGCAGGGTAACTCTAATGCTCGACTA
GCAATCAAGCAGTCCATTCATAATTTTGGCTTTATCTGGCATTGTTTCCAGGTGCTAGCTCATTATTGCTCAAGTAT
GCCCTCTCTGGTACAATCGACTCTTCCTTCTGGTATTCACTATGGAGTTAGTGTTGTGACACGAACTCTACCTTGC
TTTACTATTCTCTATCATATGTTCCATACTAATGGTGTGAAGACTATTCCTATGGATATCTTCGACTATCTTACTCCA
CCATGTCTTGCTTTTTGGATCATCGGAGATGGAACTATGGCTAATGGTGGAGGACTTTATCTACACACGCAATCCT
TTACACTAGAAGAAAATATTCGACTACTTAATGCACTAATTATTAAGTATGATATTAAGGGGGCACTACATAAGGC
GGAAGGACGTCATATTATCTATATTACAGTTCCCTCTATGCGGAAGCTATACCCTATTCTAGCACCGCATATGCATC
CTTCTATGATGTACAAGATTTTTAAGAGTATCAAGTAATAATCAAGTAATAATATAGCGGGGCAAATTCGAGAGA
ACTCCTATATATGGAATAATTAACATATATAGGACAATCGTCGAACTAAATCATTAGTTGGAAACGGCTAATGTAA
AAGCGTAGAGACTATACGCCCCAGGTCAATCTAAGTATTTATTAATATACGGTGATCTAAGACATAGTCCACTCCT
TTAAGCAATTAAAGGTAAACTCTTTTAGCCTAGATCTGAAATGATCGAATAAAGAGTAGG

(HEG Accession pfam03161, interval 239-742, E-value 1.31×10^{-49})

>BT28_COX1_INTRON3_VNIV

AAACGAAGTGCTAGGCCCACTTCATATACTGGCCAACTATATTATTACTGTTTTCGATACTATCGCAGTACCTCTTA
GCTATCTATACCCTCGTGCAACCAAGTATAAGATTCTACCTAATAATACTTGTACTAGTATTGTACCTTATGGTACC
AATCTTGGTTCTACTGTAGGGTACCCTCTATTCAATATTATTACTCGAAATATGGTAGGTATCCCTTTCAATACTCTT
TGTATTCTGGTTGGCATGATCCTTTCTGATGCTTGGATGATGAAGGACAAGCGGGGCAACTCTAATGCTCGACTA
GCAATCAAGCAATCCATTCGTAATTTTGGCTTTATCTGGCATTGTTTCCAGGTACTAGCTCATTATTGCTCAAGTAT
GCCCTCTATGGTACAATCGACTCTTCCTTCTGGTATTCACTATGGGGTAAGTGTTGTGACACGAACTCTACCTTGC
TTTACTATTCTCTATCATATGTTCCATACTAATGGTGTGAAGACTATTCCTATGGATATCTTCGACTATCTTACTCCA
CCATGCCTTGCTTTTTGGATCATGGGAGATGGAGCTATGGCTAATGGTGGAGGACTTTATCTGCACACACAATCCT
TTACACTAGAAGAAAATATTCGACTACTTAATGCACTAACTATTAAGTATGATATTAAGGTGGCACTACATAAGGC
GGAAGGACGTCATATTATTTATATTACAGTTCACTCTATGCGGAAGCTATACCCTATTCTAGCGCCACATATGCATC

CTTCTATGATGTACAAGATTTTTAAGAGTATCAAGTAATAATATAGCGGGGCAAATTCGAGAGAACTCCTGTATAT
GGAATAAGTAACATATATTGGACAATCGTCGAACTAAATCATTAGTTGGAAACGGCTAATGTAAAAGCGTAGAG
ACTATACGCCCCAGGTCAACCTAAGTATTTATTAATATACGGTGATCTAAGACATAGTCCACTCCTTTAAGCAATT
AAAGGTAGACTCTTTTAGCCTAGATCTGAAATGATCGAAGAAAGAGTAGG

(HEG Accession pfam03161, interval 236-739, E-value 5.30 × 10^{-53})

>CFP59_COX1_INTRON3_VGI

AAACGAAGTGCTAGGCCCACTTCATATACTGGCCAACTATATTATTACTGTTTTTAATATTATCACAGTACCTCTTA
ACTATCTATACCCTCGTGCGACCAAGTATAAGATTCTACCTAATAATACTTGTACTAGTATTGTACCATATGGTACC
AATCTTGGTTCGACTGTAGGGTACCCTCAATTCAATATTATTATTCGAAATATGGTAGGTATCCCTTTCAATACTCT
TTGTATTCTGGTTGGCATGATCCTTTCTGATGCTTGGATGATGAAGCACAAGCAGGGTAACTCTAATGCTCGACTA
GCAATCAAGCAGTCCATTCATAATTTTGGCTTTATCTGGCATTGTTTTCAGGTGCTAGCTCATTATTGCTCAAGTAT
GCCCTCTCTGGTACAATCGACTCTTCCTTCTGGTATTCACTATGGAGTTAGTGTTGTGACACGAACTCTACCTTGC
TTTACTATTCTTTATCATATGTTCCATACTAATGGTGTGAAGACTATTCCTATGGATATCTTCGACTATCTTACTCCA
CCATGTCTTGCTTTTTGGATCATGGGAGATGGAACTATGGCTAATGGTGGAGGGCTTTATCTACACACACAATCCT
TTACACTAGAAGAGAATATTCGACTACTTAATGCACTAATTATTAAGTATGACATTAAGGGGGCACTACATAAGG
CGGAAGGACGTCATATTATCTATATTACAGTTCCCTCTATGCGGAAGCTATATCCTATTCTAGCACCGCATATGCAT
CCTTCAATGATGTACAAGATTTTTAAGAGTATCAAGTAATAATCAAGTAATAATATAGCGGGGCAAATTCGAGAG
AACTCCTATATATGGAATAAGTAACATATATAGGACAATCGTCGAACTAAATCATTAATTGGAAACGGCTAATGTA
AAAGCGTAGAGACTATACGCCCCAGGTCAACCTAAGTATTAATTAATATACGGTGATCTAAGACATAGTCCACTC
CTTTAAGCAATTAAAGGTAGACTCTTTTAGCCTAGATCTGAAATGATCGAAGAAAGAGTAGG

(HEG Accession pfam03161, interval 239-742, E-value 2.42 × 10^{-51})

>CFP61_COX1_INTRON3_VGIII

AAACTAAGTGTTAGGTCCACTTTATTAACTGGCCAACTATATTATTAAAGTTAAATATAGCGGGGCAAATTCGAGA
GAACTCCTATATATGGAATAAGTAACATATATAGGACAATCGTCGAACTAAGTCATTAGTTGTAAACGATTAATGT
AAAAGCGTAGAGACTATACGCCCCAGGTTAACCTAAGTATTATAATAATATATGGTGATCTAAGACATAGTCCAC
TCCTTTGAGTAATTAAAGGTAGACTCTTTTAGCCTAAGTCTGAAATGATTAAATAAAGAGTAGG

>FC4_COX1_INTRON4_VNIII

ACAAATTTTGTCAAGAATGGCGCAGTACTTACTCTTGAATTTCATCTCCTGTCACGTATTAACCTACCTAATAATA
AGCCAGATGTTTCATATCTAGGTCCTTTTACAGTAGGACTGATTGATGGTGATGGTTCTATTCAGGTAAATCATTG
GCGAAGTAAGACACTACAATATCGTATTGTAGTAAAGATAGCTATTCGTCCATTTAATGAAAATATGCTGCAAAC
AATTGCTAAGACATACGGTGGTTATGTAAAGCTAGATATTAAGAACGGTTTTGTTCTGTGGATTGTAAATGACCAA
CAAACAATCCGTCAAAAGATTCTACCGCTATTTGCTCTATATCCTCCTCTCACTACACGTGTAACTCTTCAACTAG
CTTTCATGATCAAGGCCCTAAGTGGTATGACTATGGAAGAATATTTTGCTACTCGTAGTAGTAAGTATCGACAAGC
AATTACACCTCTATTTTCAACAGTACCTATTTATTTTGCTCCTTGGCTATCTGGTTTTATTGAAGCTGAAGGATCATG
GGTTCGTCGAACTTCAGTACCTTTTGGGTTTAGCTTTAGTATTGCACAATGTAATGATCTATATCTAGTTGAAGCAA
TTCTTGCTTACTTTGGCCAATCTCATCTAACGGTACAGGAACCACGAGTGCCATTCAATCGTATGCCTATTTACCA
AATTGAAATTTCAAATCGTATTGGTGTTGAAAAGGTAGTACAACATTGTCTAGATAATCCACTACTAGGATATAAG
TATTATCAACTAGCTTCAGCAATTGAAGATTCAAAGAATCTGTCACATCTGCGCCATTCTTTTTTTAAGTAAGTGT
CATGCCGTCATATCGCTATGAGAGATATTAGTTAACTCTCGGTCGATTTTTTAATTGGCTAACGGTGAAACCTTATT
CTGTCCGCTACAGACTATAAACAAGGGATAAGGCAATACCGTGGGAACCTTAAAAGGACTCCGTAGAGACTAC
ACGCGATAACGTATTAGTACGCTTCTTTTAGATGCCCGTCTAATACGTAAGATATAGTCCGATCCTGATTGTAAAA
TCAGTAACTAATCCTATTATTTTATAGGTTTTTAAAGACT

(HEG Accession pfam00961, interval 109-384, E-value 4.68 × 10^{-12})

>FC4_COX1_INTRON5_VNIII

CTGAATTGGGCCACTTAAAATCTGGTGAATTACAAGAATATCTATATTAGACAACTTGTAGCCTAGATTAGATTAG
TATTTATATAGTATAATATAATAGATCTAATAAGGTTCAACGACTAACTGGTGAGTAATACTAACAATAATCCAGA
CACGAGCGCCAGAATGCTATAATTATAGCATATGACATAGTCTAATCTGTGGTGTGAGCTACAGGATCAGGATTA
AATACTTGATCACAATATTATATGC

>BT28_COX1_INTRON4_VNIV

ACAAATTTTGTCAAGAATGGCGCAGTACTTACTCTTGAATTTCATCTCCTGTCACGTATTAACCTACCTAATAATA
AGCCAGATGTTTCATATCTAGGTCCTTTTACAGTAGGACTGATTGATGGTGATGGTTCTATTCAGGTAAATCATTG
GCGAAGTAAGACACTACAATATCGTATTGTAGTAAAGATAGCTATTCGTCCATTTAATGAAAATATGCTGCAAAC
AATTGCTAAGACATACGGTGGTTATGTGAAGCTAGATATTAAGAACGGTTTTGTTCTGTGGATTGTAAATGACCA
ACAAACAATCCGTCAAAAGATTCTACCGCTATTTGCTCTATATCCTCCTCTCACTACACGTGTAACTCTTCAACTA
GCTTTCATGATCAAGGCCCTAAGTGGTATGACTATGGAAGAATATTTTGCTACTCGTAGTAGTAAGTATCGACAAG
CAATTACACCTCTATTTTCAACAGTACCTATTTATTTTGCTCCTTGGCTATCTGGTTTTATTGAAGCTGAAGGATCAT
GGGTTCGTCGAACTTCAGTACCTTTTGGGTTTAGCTTTAGTATTGCACAATGTAATGATCTATATCTAGTTGAAGCA
ATTCTTGCTTACTTTGGCCAATCTCATCTAACGGTACAGGAACCACGAGTGCCATTCAATCGTATGCCTATTTACC
AAATTGAAATTTCAAATCGTATTGGTGTTGAAAAGGTAGTACAACATTGTCTAGATAATCCACTACTAGGATATAA
GTATTATCAACTAGCTTCAGCAATTGAAGATTCAAAGAATCTGTCACATCTGCGCCATTCTTTTTTTAAGTAAGTG
TCATGCCGTCATATCGCTATGAGAGATATTAGTTAACTCTCGGTCGATTTTTTAATCGGCTAACGGTGAAACCTTAT
TCTGTCCGCTACAGACTATAAACAAGGGATAAGGCAATACCGTGGGAACCTTAAAAGGACTCCGTAGAGACTAC
ACGCGATAACGTATTAGTACGCTTCTTTTAGATGCCCGTCTAATACGTAAGATATAGTCCGATCCTGATTGTAAAA
TCAGTAACTAATCCTATTATTTTATAGGTTTTTAAAGACT

(HEG Accession pfam00961, interval 109-384, E-value 4.68 × 10^{-12})

>BT28_COX1_INTRON5_VNIV

CTGAATTGGGCCACTTAAAATCTGGTGAATTACAAGAATATCTATATTAGACAACTTGTAGCCTAGATTAGATTAG
TATTTATATAGTATAATATAATAGATCTAATAAGGTTCAACGACTAACTGGTGAGTAATACTAACAATAATCCAGA
CACGAGCGCCAGAATGCTATAATTATAGCATATGACATAGTCTAATCTGTGGTGTGAGCTACAGGATCAGGATTA
AATACTTGATCACAATATTATATGC

>CFP59_COX1_INTRON4_ VGI

ACAAATTTTGTCAAGGATGGCGCAGTACTTACTCTTGAATTTCATCTCCTGTCACGTATTAACCTACCTAATAATA
AGCTAGATGCTTCATATCTAGGTCCTTTTACAGTAGGACTGATTGATGGTGATGGTTCTATTCAGGTAAATCATTGG
CGAAGTAAGACACTACAATATCGTATTGTAGTAAAGATAGCTATTCGTCCATTTAATGAAAATATGCTGCAAATAA
TTGCTAAGGCATACGGTGGTAATGTAAAGCTAGATACTAATAACGGTTTTGTTCTGTGGATTGTGAATGACCAAC
AAACAATCCGTCAAAAGATTCTACCCCTATTTGCTATGTATCCTCCTCTTACTACACGTGTAACTCTTCAACTAGC
TTTCATGATGAAGGCTATAAGTGGTATGACTATGGAAGAATACTTTGCTACTCGAGGTAGTAAGCTTAACAATCG
ACAAGCAATAACACCTCTATTTTCAACAGTACCTATTTATTTTGCTCCTTGGCTATCTGGTTTTATTGAAGCTGAAG
GATCATGGGTTCGTCGAATTTCAGTACCTTTCGGATTTAGCTTTAGTATTGCACAATGTCACGATCGATATCTGGTT
GAAGCTATTCTTGCTTACTTTGGCCAATCTCATCTAAAGGTACAGGTACCTCGAGTGCCTCTCAATCGTATGCCTA
TTTATCAATTTGAAATTTCAAATCGTATTGGTGTTGAAAAGGTAGTACAACATTGTATTGATAATCCACTACTAGG
ATATAAGTATTATCAACTAGCTTCAGCAATTGAAGATTCAAAGAATCTGTCACATCTGCGCCATTCTTTTTTTAAGT
AAGTGTCATGCCGTCATATCGCTATGAGAGATATTAGTTAACTCTCGGTCAGTTTTACTGGCTAACGGTGAAACCT
TATTCTGTCCGCTACAGACTATAAACAAAGGATAAGGCAATACCGTGGGAACCTTAAAAGGACTCCGTAGAGAC
TACACGCGATAACATATTATTACGCTTCTTTAGATGCCCGTCTAATATGTAAGATATAGTCCGATCCTGATTGTAAA
ATCAGTAACTAATCCCATTATTTTATAGG

(HEG Accession pfam00961, interval 109-384, E-value 5.67 × 10^{-13})

(HEG Accession cl24136, interval 508-774, E-value 7.46 × 10^{-03})

>BT27_COX1_INTRON4_VGII

ACAAATTTTGTCAAGGATGGCGCAGTACTTACTCTTGAATTTCATCTCCTGTCACGTATTAACCTACCTAATAATA
AGCTAGATGCTTCATATCTAGGTCCTTTTACAGTAGGACTGATTGATGGTGATGGTTCTATTCAGGTAAATAATTGG
CGAAGTAAGACACTACAATATCGTATTGTAGTAAAGATAGCTATTCGTCCATTTAATGAAAATATGCTGCAAATAA
TTGCTAAGGCATACGGTGGTAATGTAAAGCTAGATATTAAGAACGGTTTTGTTCTGTGGATTGTGAATGACCAAC
AAACAATCCGTCAAAAGATTCTACCTCTATTTGCTATGTATCCTCCTCTTACTACACGTGTAACTCTTCAACTAGCT
TTCATGATGAAGGCTATAAGTGGTATGACTATGGGAGAATACTTTGCTACTCGTGGTAGTAAGCTTAACGATCGA
CAAGCAATTACACCTCTATTTTCAACAGTACCTATTTATTTTGCTCCTTGGCTATCTGGTTTTATTGAAGCTGAAGG
ATCATGGGTTCGTCGAATTTCAGTACCTTTTGGATTTAGTTTTAGTATTGCACAATGTCACGATCGATATCTGGTTG
AAGCTATTCTTGCTTACTTTGGGCAATCTCATCTAAAGGTACAGGTACCTCGAGTGCCTTTCAATCGTATGCCTATT

TACCAATTTGAAATTTCAAATCGTATTGGTGTTGAAAAGGTAGTACAACATTGTCTAGATAATCCACTACTAGGAT
ATAAGTATTATCAACTAGCTTCAGCAATTGAAGATTCAAAGAATCTGTCACATCTGCGCCATTCTTTTTTTAAGTA
AGTGTCATGCCGTCATATCGCTATGAGAGATATTAGTTAACTCTCGGTCGATTTTTTATCGGCTAACGGTGAAACC
TTATTCTGTCCGCTACTGACTATAAAACAAGGGATAAGGCAATACCGTGGGAACCTTAAAAGGACTCCGTAGAG
ACTACACGCGATAACGTATTAGTACGCTTCTTTAGATGCCCGTCTAATATGTAAAATATAGTCCGATCCTGATTGTG
AAATCAGTAACTAACCCTATTATTTAATAGGTTATTAAGACT

(HEG Accession pfam00961, interval 109-384, E-value 1.07 × 10^{-11})

>CN894_COX1_INTRON4_VGII

ACAAATTTTGTCAAGGATGGCGCAGTACTTACTCTTGAATTTCATCTCCTGTCACGTATTAACCTACCTAATAATA
AGCTAGATGCTTCATATCTAGGTCCTTTTACAGTAGGACTGATTGATGGTGATGGTTCTATTCAGGTAAATAATTGG
CGAAGTAAGACACTACAATATCGTATTGTAGTAAAGATAGCTATTCGTCCATTTAATGAAAATATGCTGCAAATAA
TTGCTAAGGCATACGGTGGTAATGTAAAGCTAGATATTAAGAACGGTTTTGTTCTGTGGATTGTGAATGACCAAC
AAACAATCCGTCAAAAGATTCTACCTCTATTTGCTATGTATCCTCCTCTTACTACACGTGTAACTCTTCAACTAGCT
TTCATGATGAAGGCTATAAGTGGTATGACTATGGGAGAATACTTTGCTACTCGTGGTAGTAAGCTTAACGATCGA
CAAGCAATTACACCTCTATTTTCAACAGTACCTATTTATTTTGCTCCTTGGCTATCTGGTTTTATTGAAGCTGAAGG
ATCATGGGTTCGTCGAATTTCAGTACCTTTTGGATTTAGTTTTAATATTGCACAATGTCACGATCGATATCTGGTTG
AAGCTATTCTTGCTTACTTTGGGCAATCTCATCTAAAGGTACAGGTACCTCGAGTGCCTTTCAATCGTATGCCTATT
TACCAATTTGAAATTTCAAATCGTATTGGTGTTGAAAAGGTAGTACAACATTGTCTAGATAATCCACTACTAGGAT
ATAAGTATTATCAACTAGCTTCAGCAATTGAAGATTCAAAGAATCTGTCACATCTGCGCCATTCTTTTTTTAAGTA
AGTGTCATGCCGTCATATCGCTATGAGAGATATTAGTTAACTCTCGG

(HEG Accession pfam00961, interval 109-384, E-value 7.76 × 10^{-12})

>CN894_COX1_INTRON5_VGII

CATGGATATGACAACTTTACACTGTCTATTCAAGCACTAGGTCCTACAGACTCTGGTCAAATTTATTCACCATCAA
ACCTTATGTAGTACTAGAACAAGCCTATCTTAGCTCATACACTCTTGCTTACAATGTTAACCGAGTAGCTAGCTCA
GCCGCTTACACTCCATCTACATCGCTAGTTAATGTCGGTACTGACAACCCATCTTATGGTCTATTTGGACTAAGTT
CATATGCATGGAATAATACACATAGTGAAGCACTAAAGGCATTTTGGTCTGAGGAAAGTATAACTTCTTTGTATAT
GATAGTAGTACTCTACTACTACTACAACATTTTACTAGTGCTACTCAACTATCTAACTTCTTTGAAATGAGTAAGC
GATTTGGTACTGATATTGTAAAGAAGCTCCAAAGCCTAAACCAACCAGCACTACTATATAGTAGTTTTATTATTTC
ACTGGTAGAGCTTACAGATGCACAACTACAAGATCTGCTACCTAATATGCCAGTTAAGGCTATTATTATTCCTCGA
CCAGCTAGTCCTACAGGTAAGGTTATTTACGGCTATAACCCCGAAACTAATATTTATGAATCTTGGTCTAGTCTAG
AAAAGTGTACACATATGCTAACTGGTGCTCGATTTGCGAATAAGAGCACCGTTAATAAGCGGATTAATAAGGGA
ATTCTATTTCATGGTTATTATCTACAAACTAAGCCATTTAATAGGAGGAGTGGGCCACTTTAAATCTGGTGAATTA
CAAGAACATCTATACTCTAGACAACTTGTAGCCAAAATTAGATTAGTATTTACATACTGTAAAGGATCTAAAAAG
GTTCAACGACTAATGGGTGAGTAATACTAACAATAATCCCAACACGAGCGCCAGAATACTATATATTATAGTATAT
GACATAGTCTAATCTGTGGCGCGAGTCGCAGGATCAGAGTTAAATATCTGATCATAATATAAAGC

>FC3_COX1_INTRON4_VGIII

ACAAATTTTGTCAAGAATGGCGCAGTACTTACTCTTGAATTTCATCTCCTGTCACGTATTAACCTACCTAATAATA
AGCCAGATGTTTCATATCTAGGTCCTTTTACAGTAGGACTGATTGATGGTGATGGTTCTATTCAGGTAAATCATTG
GCGAAGTAAGACACTACAATATCGTATTGTAGTAAAGATAGCTATTCGTCCATTTAATGAAAATATGCTGCAAAC
AATTGCTAAGACATACGGTGGTTATGTGAAGCTAGATATTAAGAACGGTTTTGTTCTGTGGATTGTAAATGACCA
ACAAACAATCCGTCAAAAGATTCTACCGCTATTTGCTCTATATCCTCCTCTCACTACACGTGTAACTCTTCAACTA
GCTTTCATGATCAAGGCCCTAAGTGGTATGACTATGGAAGAATATTTTGCTACTCGTAGTAGTAAGTATCGACAAG
CAATTACACCTCTATTTTCAACAGTACCTATTTATTTTGCACCTTGGCTATCTGGTTTTATTGAAGCTGAAGGATCA
TGGGTTCGTCGAACTTCAGTACCTTTTGGGTTTAGCTTTAGTATTGCACAATGTAATGATCTATATCTAGTTGAAGC
AATTCTTGCTTACTTTGGCCAATCTCATCTAACGGTACAGGAACCACGAGTGCCATTCAATCGTATGCCTATTTAC
CAAATTGAAATTTCAAATCGTATTGGTGTTGAAAAGGTAGTACAACATTGTCTAGATAATCCACTACTAGGATATA
AGTATTATCAACTAGCTTCAGCAATTGAAGATTCAAAGAATCTGTCACATCTGCGCCATTCTTTTTTTAAGTAAGT
GTCATGCCGTCATATCGCTATGAGAGATATTAGTTAACTCTCGGTCGATTTTTTAATCGGCTAACGGTGAAACCTT
ATTCTGTCCGCTACAGACTATAAACAAGGGATAAGGCAATACCGTGGGAACCTTAAAGGACTCCGTAAAAGACT
ACACGCGATAACGTATTAGTACGCTTCTTTTAGATGCCCGTCTAATACGTAAGATATAGTCCGATCCTGATTGTAA
AATCAGTAACTAATCCTATTATTTTATAGGTTTTTAAAGACTNNNTACTATGTAGTGGCT

(HEG Accession pfam00961, interval 109-384, E-value 4.80 × 10^{-12})

>FC3_COX1_INTRON5_VGIII

CTGAATTGGGCCACTTAAAATCTGGTGAATTACAAGAATATCTATATTAGACAACTTGTAGCCTAGATTAGATTAG
TATTTATATAATATAATAGATCTAATAAGGTTCAACGACTAACTGGTGAGTAATACTAACAATAATCCAGACACGA
GCGCCAGAATGCTATAATTATAGCATATGACATAGTCTAATCTGTGGTGTGAGCTACAGGATCAGGATTAAATACT
TGATCACAATATTATATGC

>FC9_COX1_INTRON4_VGIV

ACAAATTTTGTCAAGGATGGCGCAGTACTTACTCTTGAATTTCATCTCCTGTCACGTATTAACCTACCTAATAATA
AGCTAGATGCTTCATATCTAGGTCCTTTTACAGTAGGACTGATTGATGGTGATGGTTCTATTCAGGTAAATCATTGG
CGAAGTAAGACACTACAATATCGTATTGTAGTAAAGATAGCTATTCGTCCATTTAATGAAAATATGCTGCAAATAA
TTGCTAAGGCATACGGTGGTAATGTAAAGCTAGATACTAATAACGGTTTTGTTCTGTGGATTGTGAATGACCAAC
AAACAATCCGTCAAAAGATTCTACCCCTATTTGCTATGTATCCTCCTCTTACTACACGTGTAACTCTTCAACTAGC
TTTCATGATGAAGGCTATAAGTGGTATGACTATGGAAGAATACTTTGCTACTCGAGGTAGTAAGCTTAACAATCG
ACAAGCAATAACACCTCTATTTTCAACAGTACCTATTTATTTTGCTCCTTGGCTATCTGGTTTTATTGAAGCTGAAG
GATCATGGGTTCGTCGAATTTCAGTACCTTTTGGATTTAGCTTTAGTATTGCACAATGTCACGATCGATATCTGGTT
GAAGCTATTCTTGCTTACTTTGGCCAATCTCATCTAAAGGTACAGGTACCTCGAGTGCCTCTCAATCGTATGCCTA
TTTATCAATTTGAAATTTCAAATCGTATTGGTGTTGAAAAGGTAGTACAACATTGTATTGATAATCCACTACTAGG
ATATAAGTATTATCAACTAGCTTCAGCAATTGAAGATTCAAAGAATCTGTCACATCTGCGCCATTCTTTTTTTAAGT
AAGTGTCATGCCGTCATATCGCTATGAGAGATATTAGTTAACTCTCGGTCAGTTTTACTGGCTAACGGTGAGACCT
TATTCTGTTCGCTACAGACTATAAACAAAGGATAAGGCAATACCGTGGGAACCTTAAAAGGACTCCGTAGAGAC
TACACGCGATAACATATTATTACGCTTCTTTAGATGCCCGTCTAATATGTAAGATATAGTCCGATCCTGATTGTAAA
ATCAGTAACTAACCAAGACT

(HEG Accession pfam00961, interval 109-384, E-value 5.59 × 10^{-13})

(HEG Accession cl24136, interval 508-774, E-value 8.49 × 10^{-03})
